# Engineered exosomes conferred with ROS-regulation and immuno-suppression for ameliorating lupus nephritis

**DOI:** 10.1186/s12951-025-03731-1

**Published:** 2025-10-10

**Authors:** Jiang Tian, Zexin Wang, Zhicheng Tang, Haofang Zhu, Lingyun Sun

**Affiliations:** 1 Department of Rheumatology and Immunology, Nanjing Drum Tower Hospital, Affiliated Hospital of Medical School, Nanjing University, Nanjing, China; 2 Department of Rheumatology and Immunology, The First Affiliated Hospital of Anhui Medical University, Hefei, China; 3 Department of Rheumatology and Immunology, The Second Affiliated Hospital of Anhui Medical University, Hefei, China

**Keywords:** MSCs, Exosomes, Ceria, Lupus nephritis, Homing

## Abstract

Mesenchymal stromal cell-derived exosomes (MEXs) possess inherent homing capabilities, tissue repair abilities, and anti-inflammatory and immunomodulatory functions, making them a promising alternative for treating lupus nephritis (LN). In this study, we present an innovative engineered MEX that serves both as a reactive oxygen species (ROS) scavenger and a rapamycin carrier for targeted LN therapy. The nanohybrid (CEX@Rapa) was developed by chemically conjugating ceria (Ce) nanoparticles to rapamycin-loaded thiol-functionalized MEXs through a Michael addition reaction. The resulting nanohybrids demonstrate minimal toxicity, optimal drug loading efficiency, superior cellular uptake capability, and remarkable anti-oxidant, anti-damage, and anti-inflammatory properties. Upon systemic administration to MRL/lpr mice, these nanohybrids tend to accumulate and persist at the site of kidney inflammation, mitigating the excessive immune response and promoting kidney repair, leading to significant alleviation of LN symptoms through the independent and synergistic effects of MEXs, Ce nanoparticles, and rapamycin. Therefore, we believe that Ce-immobilized MEXs, as an enhanced drug nanocarrier with ROS-regulating and immunomodulatory capabilities, hold substantial promise for treating LN and other autoimmune diseases.

**Graphical Abstract:**

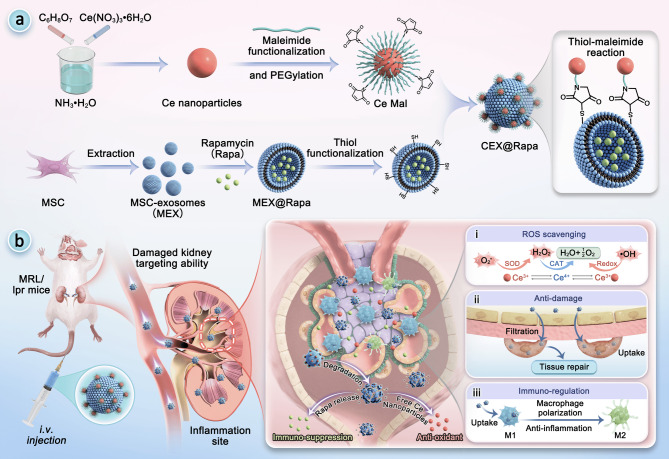

**Supplementary Information:**

The online version contains supplementary material available at 10.1186/s12951-025-03731-1.

## Introduction

Systemic lupus erythematosus (SLE) is a classic autoimmune disorder characterized by chronic inflammation across multiple organs, with lupus nephritis (LN) being a predominant and severe manifestation that causes significant distress among affected individuals [1–5]. Corticosteroids and immunosuppressants, particularly rapamycin, are commonly employed in clinical practice for the management of LN [6–8]. While rapamycin effectively suppresses the immune response to achieve disease remission, high doses and systemic administration often result in rapid metabolism and off-target distribution, leading to unavoidable and severe adverse effects, including increased susceptibility to infections and hepatic impairment [9, 10]. To minimize administration frequency and mitigate side effects, various advanced nanomedicine approaches utilizing synthetic materials, such as liposomes, micelles, and polymer assemblies have been developed to enhance LN treatment efficay [11–13]. Despite some advancements, the clinical application of these nanocarriers remains limited due to challenges such as low drug loading capacity, poor stability, lack of multifunctionality, and potential toxicity [14–17]. Therefore, there is an urgent need to develop a multipotent nanomedicine with improved properties for the effective treatment of LN.

**Fig. 1:**
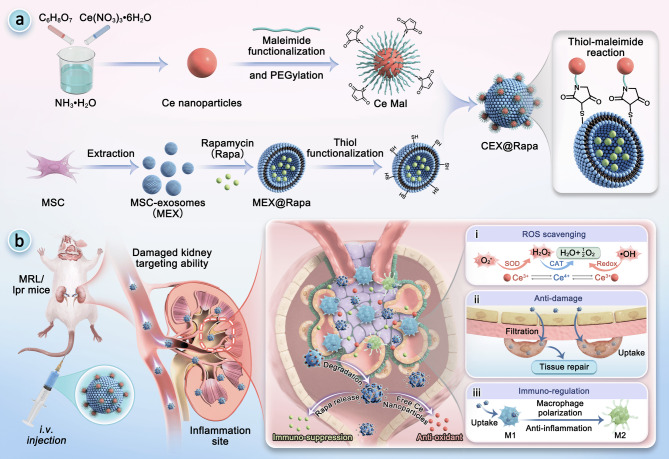
Schematic of renal-targeting rapamycin-loaded/ROS-scavenging nanohybrid for LN therapy. (**A**) Preparation of CEX@Rapa. (**B**) CEX@Rapa migrated to injured kidney because of its homing ability, releasing Ce nanoparticles and rapamycin in response to ROS. Then, CEX@Rapa performed (i) ROS scavenging, (ii) anti-damage and (iii) immuno-regulation through the independent and synergistic effects of MEXs, Ce nanoparticles, and rapamycin

In this study, we introduced a new nanohybrid designed to target inflamed kidneys, serving as both a rapamycin carrier and a reactive oxygen species (ROS) scavenger for LN treatment, as illustrated in Fig. 1. Exosomes, which are nanovesicles secreted by diverse cell types, significantly contribute to cell-cell interactions that influence the onset and progression of various diseases [18–20]. Among these native exosomes, those derived from mesenchymal stromal cells (MSCs) have attracted increasing attention due to their inherent homing capabilities, tissue repair abilities, and anti-inflammatory and immunomodulatory functions [21–23]. Compared to direct MSCs transplantation, MSCs-derived exosomes (MEXs) offer enhanced stability, reduced toxicity, and the ability to deliver immunomodulatory cytokines to target cells [24–27]. On the other hand, ceria (Ce) nanoparticles function as antioxidants by effectively scavenging ROS through reversible cycling between two ionic states, Ce^3+^ and Ce^4+ ^[28–31]. This mechanism enables Ce nanoparticles to mitigate excessive ROS production at inflamed sites and promotes M1-to-M2 macrophage polarization, alleviating inflammation and associated symptoms [32, 33]. Therefore, integrating MEXs and Ce nanoparticles to develop multi-functional rapamycin nanocarriers holds great promise for alleviating the severe complications of LN.

In this paper, we developed a Ce nanoparticle immobilized MEX loaded with rapamycin (CEX@Rapa) nanohybrid system for targeted therapy in LN. Rapamycin was physically encapsulated into MEXs via ultrasonication, and CEX@Rapa was subsequently synthesized through a thiol-maleimide reaction between maleimide-functionalized Ce nanoparticle (Ce-Mal) and thiol-functionalized MEXs. The resultant CEX@Rapa demonstrated stable particle size and zeta potential, optimal drug loading efficiency, sustained drug release, minimal toxicity, and excellent ROS-scavenging activities. Moreover, both mouse podocyte clone-5 (MPC-5) and macrophages were shown to specifically endocytose CEX@Rapa, indicating the presence of dual-targeting effects. At the cellular level, CEX@Rapa exhibits anti-inflammatory and anti-damage activities. In vivo fluorescence imaging confirmed that the nanohybrid could specifically accumulate and maintain prolonged retention in inflamed renal tissues. In the SLE models, the three key elements of the nanohybrid (MEXs, Ce nanoparticles, and rapamycin) work both independently and synergistically to mediate anti-apoptosis in podocyte injury, modulate the excessive systemic immune response, provide rapid treatment of the damaged glomerular basement membrane (GBM), and repair kidney damage. These results indicated that the proposed ceria-immobilized MEXs as a drug delivery nanohybrid is a promising strategy for accurate guidance of therapeutics against LN.

## Results and discussion

### Design, fabrication and characterization of CEX@Rapa

**Fig. 2:**
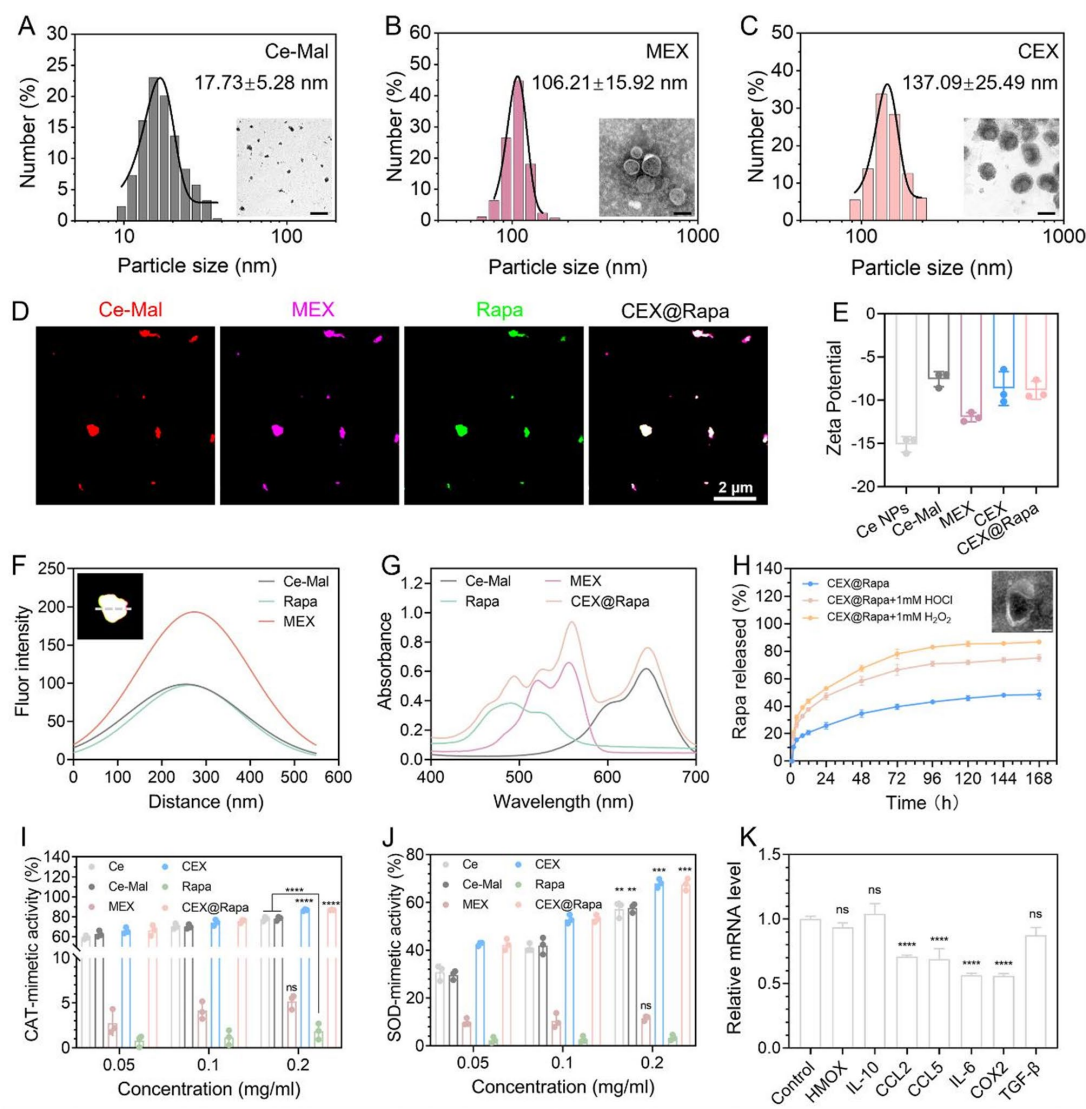
Characterization of CEX@Rapa nanohybrid. (**A**-**C**) Hydrodynamic size distribution and TEM images of Ce-Mal, MEX, and CEX. Scale bar: 100 nm (**D**) The co-localization of Cy5-labeled Ce-Mal (red), DiO-labeled MEX (violet), and Cy2-labeled rapamycin (green) shown in super-resolution microscopy images. (**E**) ζ-potential measurements for Ce nanoparticles, Ce-Mal, MEX, CEX, and CEX@Rapa. (**F**) The fluorescence intensities for CEX@Rapa tagged with Cy5 (red, Ce-Mal), DiO (violet, MEX), and Cy2 (green, rapamycin). (**G**) UV absorbance spectra of Cy5-labeled Ce-Mal, DiO-labeled MEX, Cy2-labeled rapamycin, and CEX@Rapa, constructed from Cy5-labeled Ce-Mal, DiO-labeled MEX, and Cy2-labeled rapamycin. (**H**) ROS-responsive release profiles of rapamycin from CEX@Rapa and corresponding exosome membrane integrity. The inset shows representative TEM images of exosomes after 24-hour incubation with 1 mM H₂O₂, revealing membrane disruption. Scale bar: 100 nm. (**I**, **J**) Anti-oxidant effects evaluation: CAT- (**I**) and SOD-mimicking activity (**J**) for Ce nanoparticles, Ce-Mal, MEX, CEX, rapamycin, and CEX@Rapa. (**K**) qRT-PCR analysis of cytokines in MEX and CEX@Rapa. Data are presented as mean ± standard deviation (*n* = 3). Statistical significance was evaluated relative to the Rapa group (**I**, **J**) and the Control group (**K**) using one-way ANOVA and Tukey’s post hoc test. **P < 0.01, ***P < 0.001, ****P < 0.0001. ns, not significant

Uniformly sized Ce nanoparticles were synthesized via the reaction of cerium acetate hydrate and citric acid (Figure S1). X-ray photoelectron spectroscopy (XPS) analysis confirmed the coexistence of cerium in mixed valence states (Ce³⁺ and Ce⁴⁺) on the nanoparticle surface (Figure S2). The calculated Ce³⁺/Ce⁴⁺ ratio of approximately 1.30 indicates a high concentration of oxygen vacancies, which contributes to robust ROS scavenging capacity. Furthermore, we evaluated the potential ROS scavenging ability of Ce nanoparticles, specifically targeting superoxide anion (O_2_^−^) and hydrogen peroxide (H_2_O_2_), which are key ROS that can cause oxidative stress and cell apoptosis when overproduced [30]. Electron paramagnetic resonance (EPR) measurements showing a scavenging rate of 78.76% for the O₂⁻ (Figure S3A). In the case of H₂O₂, spectral line broadening and reduced hyperfine resolution indicated an elevated oxygen concentration, confirming effective decomposition (Figure S3B). Then, Ce nanoparticles were covered with DSPE-PEG and DSPE-PEG-MAL to enable water dispersion and maleimide modification, producing Ce-Mal. Dynamic light scattering (DLS) analysis revealed that Ce-Mal had an average size of approximately 17.73 nm with a polydispersity index (PDI) of 0.3174 (Fig. 2A).

To obtain the MEXs, MSCs at passages 3 to 6 were cultured extensively in serum-free medium. The cell culture media were collected after 48 h and subjected to ultracentrifugation at 100,000 × g for MEX isolation [27]. The quality control parameters of exosomes obtained from different batches demonstrated consistent compliance with established standards (Table S1). According to transmission electron microscopy (TEM), the MEXs demonstrated a distinct bilayer membrane microstructure, characteristic of typical extracellular vesicles (Fig. 2B, and S4). The presence of exosome markers (CD81, TSG101 and Alix) and the cell contamination marker (calnexin and histone H1) was confirmed (Figure S5). These findings demonstrate that MEXs were successfully isolated from the supernatants of MSC cultures for subsequent experiments.

The disulfide bond on the MEX membrane was disrupted by Tris (2-carboxyethyl) phosphine (TCEP), exposing the sulfhydryl groups. The Ce nanoparticle-immobilized MEX (CEX) was then constructed by a Michael addition reaction, using Ce-Mal as the decorative component and thiol-functionalized MEX as the support. TEM images reveal that numerous Ce nanoparticles densely coated the surface of MEXs, leading to an increase in hydrodynamic diameter as they attached (Fig. 2C and S6).

To produce the drug-loaded CEX (CEX@Rapa), rapamycin was physically encapsulated into thiol-functionalized MEXs through ultrasonication, followed by conjugation with Ce-Mal via a thiol-maleimide reaction. The successful construction of CEX@Rapa was confirmed by examining the co-localization of Ce-Mal, MEXs, and rapamycin (Fig. 2D, F and G, and S7). After Ce nanoparticles bonded to the MEX surface, ζ-potential measurements of the CEX and CEX@Rapa indicated a reduction in negative charge (Fig. 2E). We then evaluated the drug loading content of rapamycin in CEX@Rapa, which was 23.66%, and the drug loading efficiency, which was 60.6%. UV spectra demonstrated that CEX encapsulation significantly enhanced the stability of free rapamycin, with only 50% degradation observed after 15 days of incubation in PBS at pH 7.4 (Figure S8). The release profile of rapamycin from CEX@Rapa exhibited sustained and slow kinetics under physiological conditions (PBS, pH 7.4), with approximately 40% of the drug released over 120 h. In contrast, under pathologically relevant oxidative conditions (1 mM H₂O₂ or 1 mM HOCl), release was significantly accelerated, reaching approximately 80% and 70% respectively within the same period—nearly twice that of the physiological control (Fig. 2H). This enhanced release under oxidative conditions is attributed to ROS-induced structural disruption of the exosomal membrane. Specifically, as confirmed by TEM imaging (inset, Fig. 2H), treatment with 1 mM H₂O₂ for 24 h resulted in clear loss of membrane integrity and vesicle deformation, consistent with lipid peroxidation-mediated breakdown [34–36]. This mechanism enables targeted rapamycin release within inflammatory sites characterized by high ROS levels, while minimizing premature leakage under normal physiological conditions, highlighting its suitability for delivering drugs to the oxidative renal environment associated with LN [37].

To evaluate the therapeutic efficacy of CEX@Rapa, its catalytic performance was investigated first. In managing inflammatory diseases, a primary objective is the rapid removal of these species to alleviate inflammation and cytokine release. Owing to the outstanding ROS-scavenging abilities of the Ce nanoparticles, CEX@Rapa demonstrated effective catalase (CAT) and superoxide dismutase (SOD) mimicking activities. (Fig. 2I and J). Notably, MEX showed reduced activity levels, probably because MSCs secrete SOD [38]. Additionally, the antioxidant capabilities of Ce and Ce-Mal nanoparticles showed minimal differences. These results suggest that the chemical modification with MEX did not affect the catalytic activities of the Ce nanoparticles.

We then investigated if CEX@Rapa retains the properties of MEXs, which are known for their anti-inflammatory and immunomodulatory properties [39]. The presence of key immunomodulatory elements (IL-10, TGF-β, and COX-2) in CEX@Rapa confirmed that these therapeutic agents were maintained during manufacturing (Fig. 2K). Additionally, we observed reduced levels of pro-inflammatory genes (IL-6, CCL-2, CCL-5, and HMOX), indicating that the physical encapsulation of rapamycin and the chemical immobilization of Ce nanoparticles had a synergistic anti-inflammatory effect. These results suggest that CEX@Rapa can exert immunomodulatory properties and perform catalytic functions through transporting therapeutic molecules to specific cells.

### Anti-inflammatory and Immunomodulatory effects of CEX@Rapa

RAW 264.7 macrophages were utilized for assessing the biocompatibility of CEX@Rapa prior to biological evaluation, and no apparent toxicity was found over 72 h of co-incubation (Figure S9). In contrast, only 60% of cells survived in the free rapamycin group, indicating that MEX encapsulation significantly reduced the cytotoxicity of rapamycin.

**Fig. 3:**
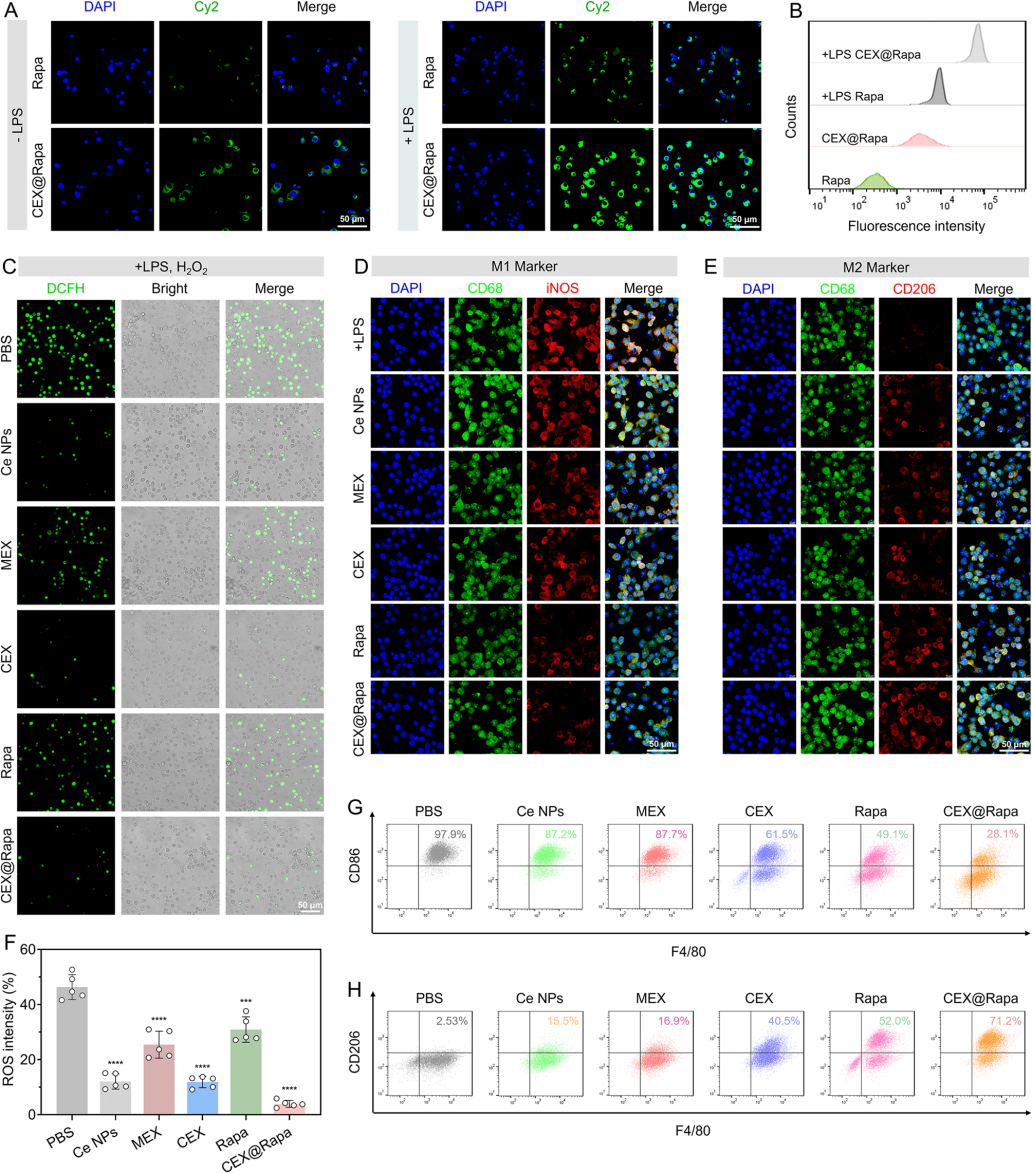
Anti-inflammatory and immune-modulating effects of CEX@Rapa on macrophages (M1 to M2 transition). (**A**) Fluorescent images and (**B**) flow cytometry analysis of inactivated and activated RAW 264.7 cells treated with Cy2-labeled rapamycin and CEX@Rapa for 2 h. (**C**) Confocal microscopy verifying the ROS-scavenging activity of CEX@Rapa in vitro, with related quantifications shown in (**F**). (**D**, **E**) Immunofluorescent labeling highlights the macrophage profile, showing iNOS (red, M1 indicator), CD206 (red, M2 indicator), CD68 (green, general macrophage indicator), and cell nuclei (blue). (**G**, **H**) The phenotypic transition from M1 to M2 in macrophages revealed by flow cytometry analysis. Data are presented as mean ± standard deviation (*n* = 5), with statistical significance evaluated relative to the PBS group using one-way ANOVA and Tukey’s post hoc test. ***P < 0.001, and ****P < 0.0001

We performed a macrophage uptake assay to further assess the effectiveness of the prepared nanohybrids. Macrophages are essential in the secretion of inflammatory mediators and are plentifully found at sites of inflammation [40]. Hence, assessing the uptake of CEX@Rapa by activated macrophages is essential for determining its effectiveness in treating LN. Minimal free rapamycin was endocytosed by both LPS activated and non-activated macrophages (Fig. 3A). In contrast, significant cellular uptake of CEX@Rapa was observed in activated macrophages (Fig. 3B), indicating that exosomes enhance drug ingestion, enabling macrophages to effectively take up the drugs and exert their therapeutic effects.

To elucidate the mechanistic role of the loaded rapamycin, we evaluated its impact on mTOR pathway inhibition and autophagy induction in LPS-activated RAW 264.7 macrophages via immunofluorescence. As shown in Figure S10 and S11, treatment with CEX@Rapa markedly reduced the fluorescence signals of phosphorylated S6K (p-S6K) and 4EBP1 (p-4EBP1), key downstream effectors of mTORC1, confirming effective suppression of mTOR signaling. Concurrently, we observed a significant increase in LC3B expression, indicative of autophagosome formation, accompanied by a notable decrease in p62 expression, reflecting enhanced autophagic flux and robust autophagy activation.

Given that ROS overproduction primarily triggers macrophage activation, we explored the ability of nanohybrids to scavenge ROS in vitro. The findings from confocal microscopy indicated that CEX@Rapa successfully eliminated the excess ROS in activated macrophages, with ROS intensity levels comparable to those of Ce nanoparticles and CEX alone, highlighting the Ce-driven ROS removal capability (Fig. 3C and F). The combination of mTOR-dependent autophagy activation and Ce-mediated ROS clearance creates a coordinated intracellular environment conducive to anti-inflammatory polarization.

Considering this, we further evaluated the polarization of macrophages from the pro-inflammatory M1 phenotype to the anti-inflammatory M2 phenotype under inflammatory conditions. M1 macrophages, characterized by high iNOS expression, exacerbate SLE progression through the secretion of inflammatory cytokines [37]. Transitioning to the M2 phenotype (marked by CD206) represents a promising therapeutic strategy for LN. In the CEX@Rapa treatment group, we observed significant enrichment of CD206 and downregulation of inflammation-related proteins compared to the PBS control (Fig. 3D and E). Flow cytometry analysis confirmed a pronounced shift from M1 to M2 polarization (Fig. 3G, H and Figure S12). Although individual treatments with Ce nanoparticles, MEX, or rapamycin alone exhibited anti-inflammatory effects, CEX@Rapa demonstrated superior efficacy, suggesting synergistic cooperation among the immunomodulatory cargo of MEX, rapamycin-mediated immunosuppression (via mTOR inhibition and autophagy induction), and Ce nanoparticle-driven catalytic activity in modulating innate immune responses. The MEX and rapamycin addresses the inflammatory signaling cascades from within, while the Ce nanoparticles mitigates the external oxidative stress that fuels inflammation, resulting in a more robust and sustained anti-inflammatory outcome.

### Antioxidant and anti-damage activity of CEX@Rapa

**Fig. 4:**
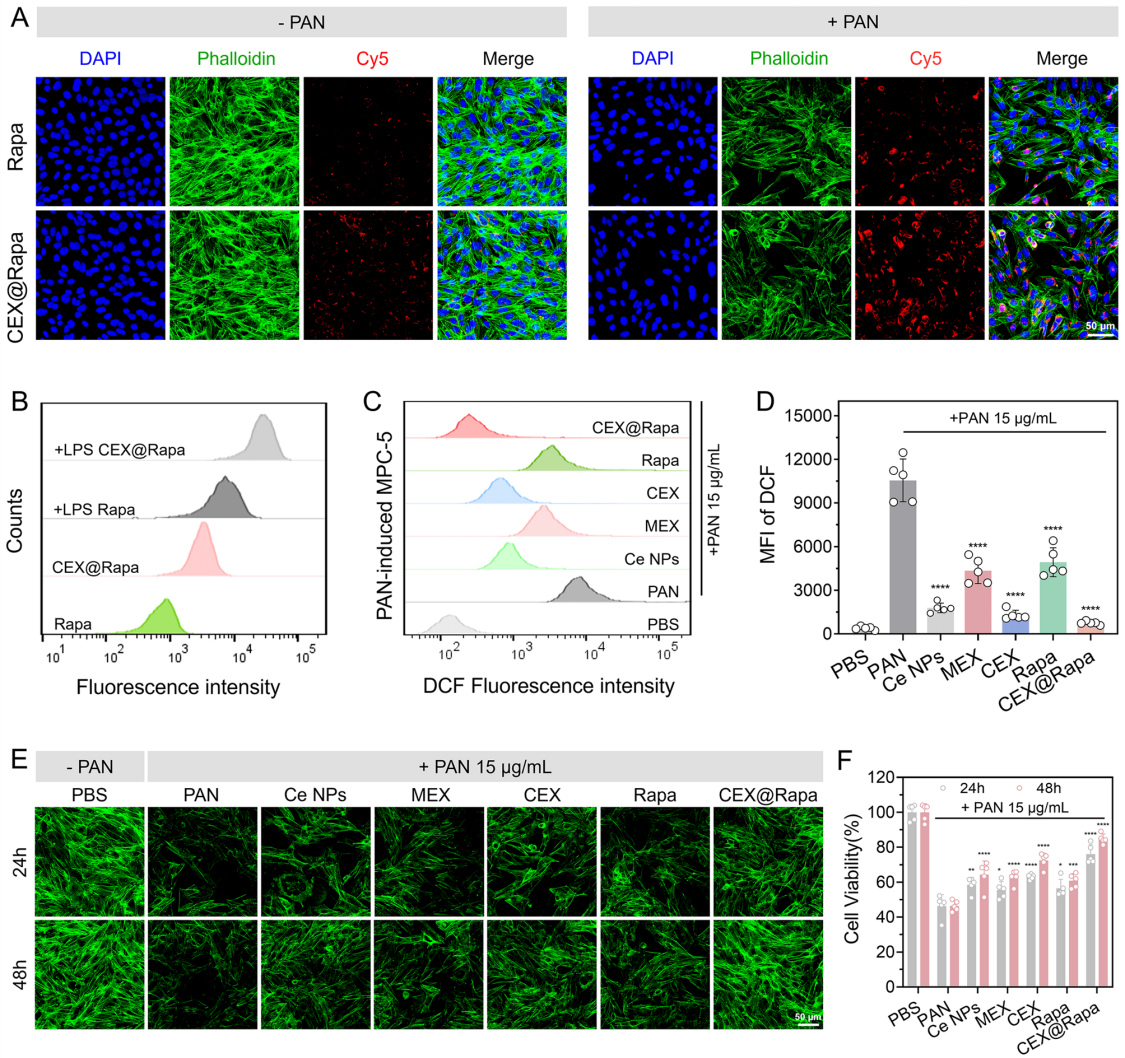
Antioxidant and anti-apoptosis effect of CEX@Rapa in podocytes. (**A**) Fluorescent images and (**B**) flow cytometry analysis of normal and PAN-induced MPC-5 cells treated with Cy5-labeled rapamycin and CEX@Rapa for 2 h. (**C**) ROS levels measured by flow cytometry following DCFH-DA dyeing. (**D**) Statistical representation for mean DCF fluorescence intensity. (**E**) Fluorescent images and morphology of MPC-5 cells showing cell repair in 24 h and 48 h. (**F**) CCK-8 assessed cell viability. Data are presented as mean ± standard deviation (*n* = 5), with statistical significance evaluated relative to the PAN group using one-way ANOVA and Tukey’s post hoc test. *0.01 < *P* < 0.05, **0.001 < *P* < 0.01, ***P < 0.001, and ****P < 0.0001

Podocytes and their foot processes play a crucial role in glomerular filtration. Damage to these structures is crucial for the development of nephrotic syndrome associated with various glomerular injuries [41]. The biocompatibility of CEX@Rapa was assessed using MPC-5 cells, and no evident toxicity was observed (Figure S13**).** Given that nanohybrids can enter the bloodstream and target damaged kidney tissues for drug delivery due to the inherent ability of stromal cell-derived exosomes to target inflamed sites [22]. We investigated the uptake efficiency of CEX@Rapa using the podocyte cell line MPC-5. For this, puromycin amino nucleotide (PAN) was administered at a concentration of 15 µg/mL to establish a podocyte injury model. As shown in Fig. 4A, we observed a substantial accumulation of fluorescent nanohybrids in PAN-induced damaged podocytes. Quantitative data further confirmed an increase in fluorescence intensity in the MPC-5 injury model, as determined by flow cytometry (Fig. 4B), indicating that CEX@Rapa is readily phagocytized by podocytes, thereby facilitating its therapeutic effects.

Intracellular ROS levels in PAN-induced podocytes were analyzed and quantified using the DCFH-DA probe. The results revealed that PAN significantly elevated ROS levels in podocytes, while Ce nanoparticles and CEX effectively reduced these levels (Fig. 4C and D). Notably, free rapamycin could partially reverse the oxidative damage in podocytes, likely by activating autophagy. Among the treatments, CEX@Rapa exhibited the highest scavenging capacity in renal cells, attributed to the synergistic antioxidant effects of Ce nanoparticles and the released rapamycin. This discovery aligns with the in vitro release behavior of CEX@Rapa, which facilitates the cleavage and release of rapamycin under conditions of high ROS concentration (Fig. 1H).

We next assessed the damage-repair of PAN-induced podocytes to determine whether CEX@Rapa could repair cell tissue. Our results indicated that PAN induced podocyte injury in a time-dependent manner, as demonstrated by cell actin staining and the CCK-8 assay (Fig. 4E and F). The increase in cell area further confirmed the anti-damage effects of CEX@Rapa on MPC-5, leading to the rearrangement of the podocyte actin cytoskeleton and enhanced adhesion of the repaired podocytes. It is noted that rapamycin alone significantly improved or even reversed PAN-induced podocyte injury, likely through the rapamycin-mediated inhibition of the PI3K/AKT/mTOR signaling pathway and autophagy [42, 43]. In summary, these data highlight the anti-damage and antioxidant activities of CEX@Rapa, which are essential for podocyte and renal tissue repair.

### CEX@Rapa targets and accumulates in the kidney of MRL/lpr mice

**Fig. 5:**
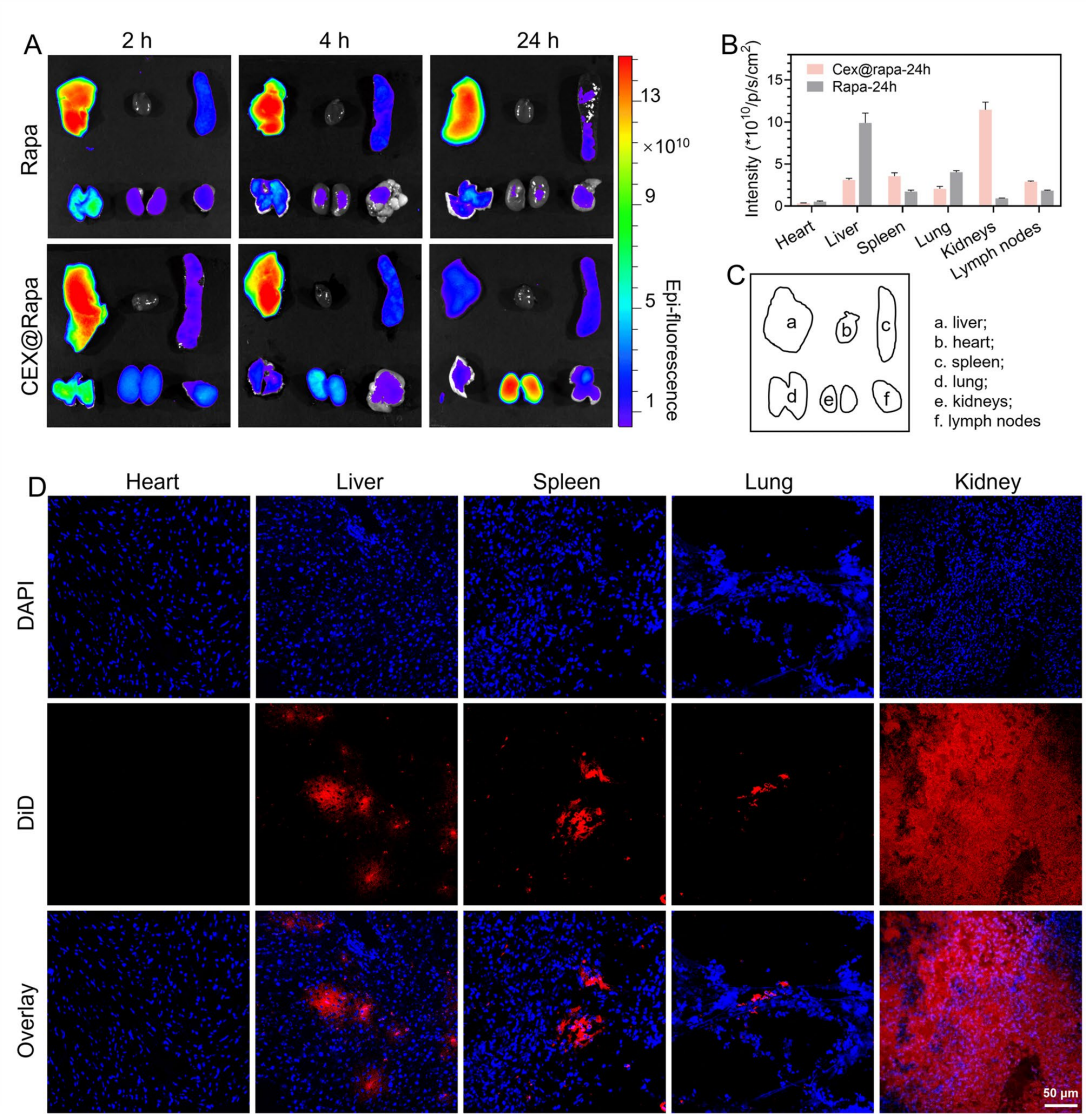
Therapeutic mechanisms of nanohybrids in treating LN. (**A**) The biodistribution of CEX@Rapa in primary isolated organs was assessed using NIRF imaging at 2, 4, and 24 h after injection. (**B**) The average NIRF intensity of nanohybrids was quantified in MRL/lpr mice 24 h after i.v. administration. (**C**) (a) liver; (b) heart; (c) spleen; (d) lung; (e) kidneys; (f) lymph nodes. (**D**) In the CEX@Rapa group, major organs were preserved, sectioned, and stained with DAPI 24 h following nanohybrids administration. The localization of nanohybrids is demonstrated in immunofluorescence images. Blue represents DAPI staining, while red indicates DiD-labeled nanohybrids

A live animal imaging system was employed to evaluate the targeting efficacy of CEX@Rapa. DiD-tagged CEX@Rapa was administered intravenously (*i.v.*) to MRL/lpr mice, and its distribution was monitored using the live animal imaging system. Compared to free rapamycin, CEX@Rapa showed a marked buildup in the kidneys at 2, 4, and 24 h following administration (Fig. 5A). Notably, the near-infrared fluorescence (NIRF) signal in CEX@Rapa remained robust 24 h after administration, while the NIRF intensity treated with free rapamycin decreased considerably during the same period. This finding highlights the enhanced targeting accuracy and retention conferred through exosome encapsulation (Fig. 5B, C, and S14).

Furthermore, tissue sections immunofluorescence analysis verified the selective renal targeting capability of CEX@Rapa. The inflamed kidneys showed high fluorescence intensity, whereas the heart, liver, spleen, and lungs of MRL/lpr mice had minimal fluorescence accumulation (Fig. 5D). This targeted delivery to damaged kidneys is probably attributed to the increased permeability of renal blood vessels and the inherent capacity of stromal cell-derived exosomes to localize at inflamed sites. Overall, these results demonstrate CEX@Rapa’s promise as a therapeutic agent for renal injury by utilizing both its targeted delivery and sustained retention in affected tissues.

### CEX@Rapa relieves LN progression in MRL/lpr mice

**Fig. 6:**
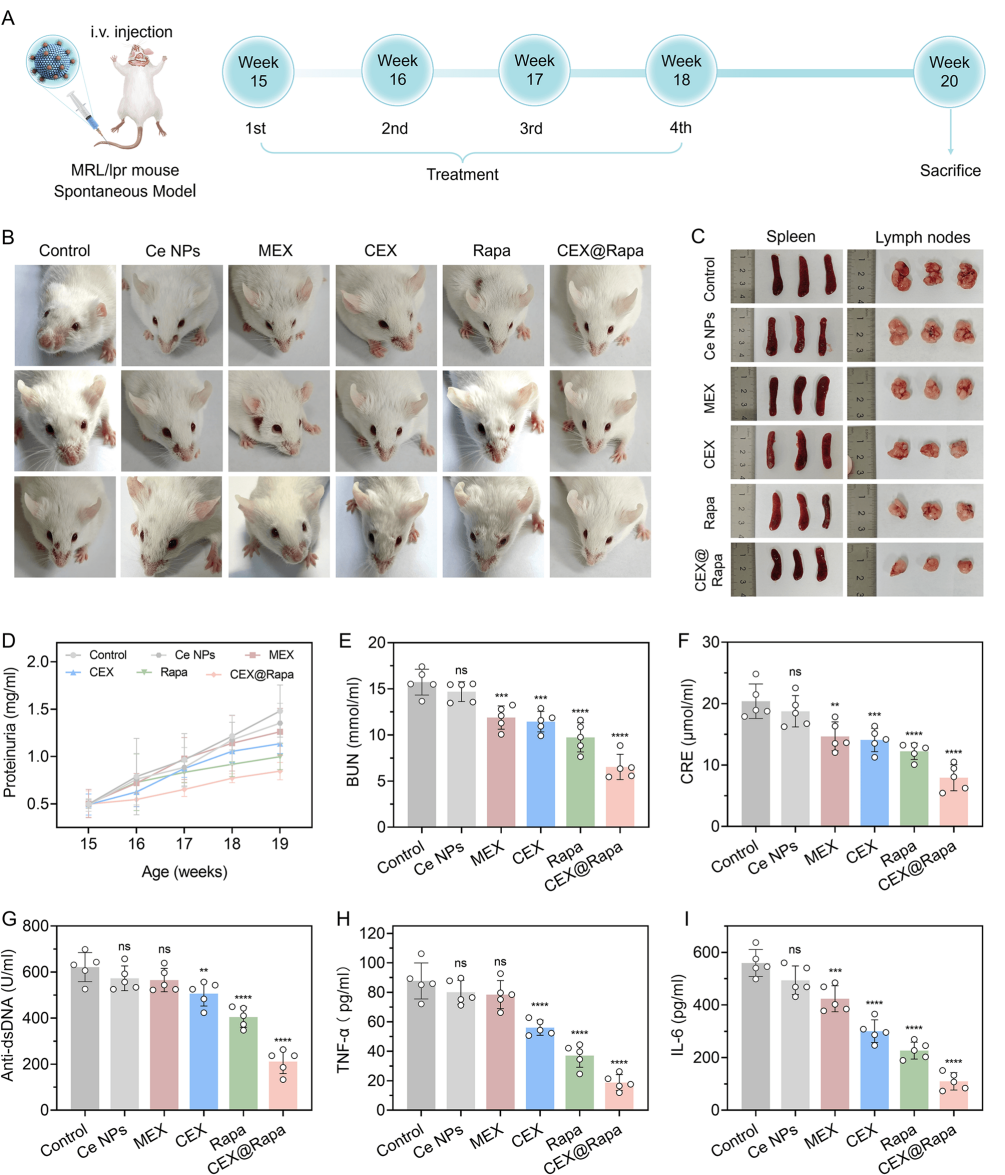
The symptoms of LN are alleviated by CEX@Rapa. (**A**) Illustration of the treatment regimen for MRL/lpr mice. (**B**) Contrast in the severity of facial lesions among various groups at the 20th week. (**C**) Comparative visualization of spleens and lymph nodes across different groups. (**D**) The body weight of MRL/lpr mice in the process of treatment by Control, Ce nanoparticles, MEX, CEX, rapamycin, and CEX@Rapa. (**E**) assessment of BUN as an extra marker for kidney function. (**F**) Evaluation of kidney function by measuring serum creatinine. (**G**) Anti-dsDNA Abs levels. (**H**, **I**) Cytokines TNF-α and IL-6 in Control, Ce nanoparticles, MEX, CEX, rapamycin, and CEX@Rapa groups were measured by ELISA. Data are presented as mean ± standard deviation (*n* = 5), with statistical significance evaluated relative to the Control group using one-way ANOVA and Tukey’s post hoc test. **0.001 < *P* < 0.01, ***P < 0.001, ****P < 0.0001. ns, not significant

CEX@Rapa’s therapeutic potential in vivo was evaluated by intravenously administering it to MRL/lpr mice, recognized for SLE. The treatment plan included weekly i.v. administration of saline, Ce nanoparticles, MEX, CEX, rapamycin, and CEX@Rapa over a four-week period, focusing on LN (Fig. 6A). Notably, the group treated with CEX@Rapa showed a significant reduction in facial lesions area (mean ± SEM: 3.39% ± 0.68%) compared with alternative treatment groups (Fig. 6B and S15, S16). Additionally, this group showed considerably reduced spleen and lymph node sizes, supported by weight measurement comparisons (Fig. 6C and S17). Throughout the treatment period, MRL mice exhibited stable body weight maintenance with consistent growth trends (Figure S18).

To assess kidney dysfunction-an important indicator in LN-we analyzed the effects of CEX@Rapa on essential markers of glomerular filtration in MRL/lpr mice, such as proteinuria, blood urea nitrogen (BUN) and creatinine levels (Fig. 6D, E and F). Our findings revealed that CEX@Rapa nanohybrids outperformed all other groups by significantly reducing proteinuria, creatinine and BUN levels. In SLE, affected animals typically exhibit elevated levels of anti-dsDNA antibodies, which are strongly linked to glomerulonephritis. According to Fig. 6G, MRL/lpr mice given either Control, Ce nanoparticles, MEX, CEX, or rapamycin alone displayed higher anti-dsDNA antibody levels. Conversely, mice that received CEX@Rapa showed a marked decrease in anti-dsDNA Abs levels. In addition, ELISA findings revealed that the CEX@Rapa group exhibited notably decreased levels of inflammatory cytokines TNF-α and IL-6 after treatment, compared with alternative groups (Fig. 6H and I), indicating a notable reduction in the production of inflammatory cytokines in MRL/lpr mice. These outcomes corporately affirm the sustained synergistic effect of CEX@Rapa hybrid system in the progression of LN and highlight its potential for treating other conditions associated with immune dysregulation.

**Fig. 7:**
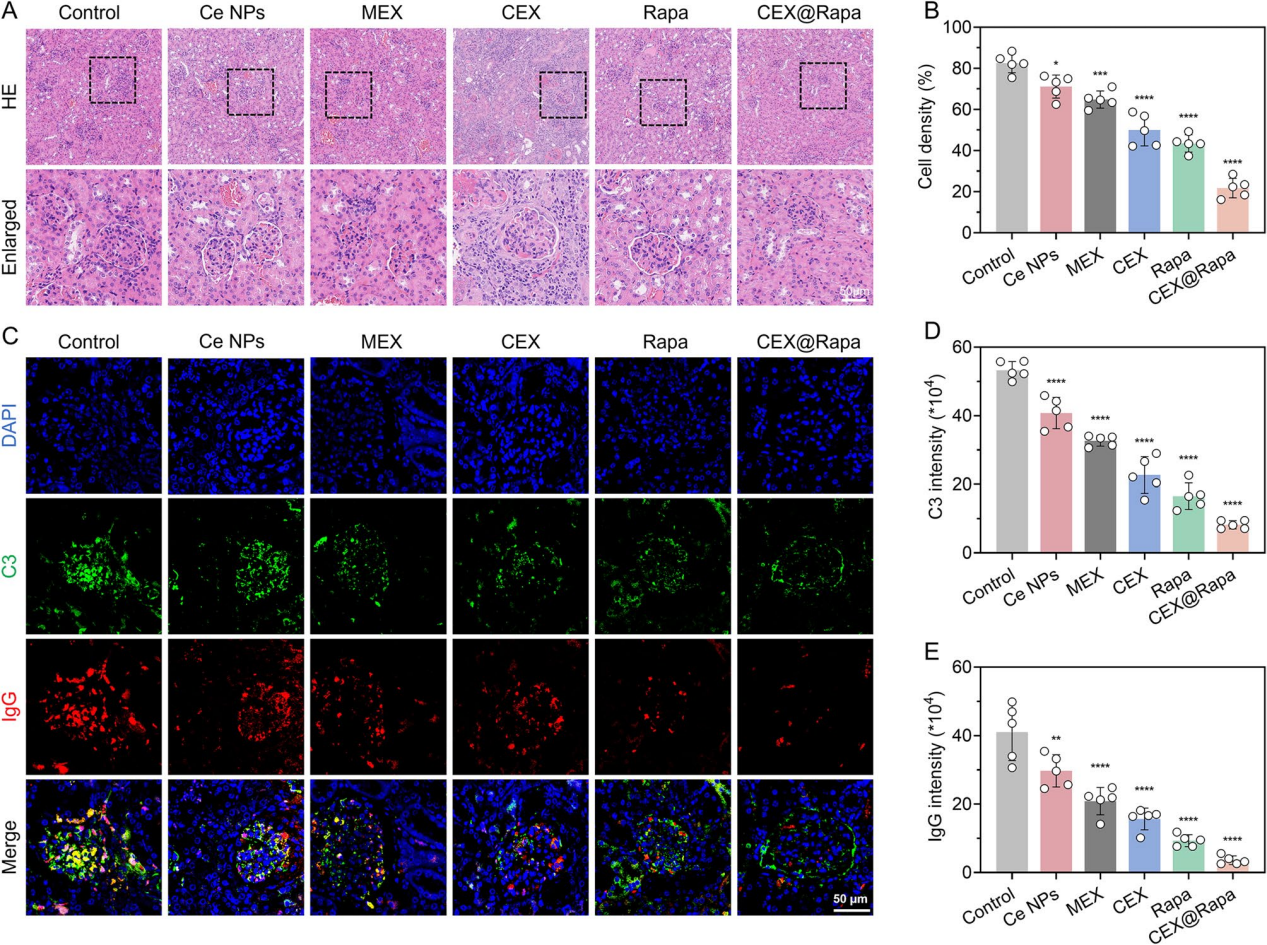
Histological examination of LN following CEX@Rapa therapy. (**A**) Comparison of H&E staining in renal sections among Control, Ce nanoparticles, MEX, CEX, rapamycin, and CEX@Rapa groups. (**B**) Measurement of the overall density of mesangial cells. (**C**) C3, and IgG immunofluorescent labeling in renal sections among Control, Ce nanoparticles, MEX, CEX, rapamycin, and CEX@Rapa groups. The fluorescence intensity of (**D**) C3 and (**E**) IgG was quantitatively analyzed using Image J software. Data are presented as mean ± standard deviation (*n* = 5), with statistical significance evaluated relative to Control group using one-way ANOVA and Tukey’s post hoc test. *0.01 < *P* < 0.05, **0.001 < *P* < 0.01, ***P < 0.001, ****P < 0.0001

Spontaneous renal complications, like membranoproliferative glomerulonephritis (MPGN), commonly occur in MRL/lpr mice and are characterized by the accumulation of C3 and IgG in the glomeruli. According to the histological analysis, CEX@Rapa effectively lowered glomerular issues, evidenced by limited cell invasion and decreased mesangial hyperplasia (Fig. 7A and B). Additionally, the CEX@Rapa treatment effectively diminished the accumulation of immune complexes, as evidenced by lower levels of C3 and IgG staining (Fig. 7C-E). While the clinical application of rapamycin and Ce nanoparticles is often limited by their intrinsic toxicity, the challenge lies in mitigating these adverse effects without compromising their efficacy. Notably, the CEX@Rapa not only attenuated LN progression effectively but also exhibited minimal organ toxicity in vivo. Specifically, serum levels of ALP, ALT, and AST in treated MRL/lpr mice remained within normal physiological ranges, indicating an absence of hepatotoxicity-a finding consistent with prior in vitro biosafety assessments (Figure S19 and S20).

These findings highlight the synergistic effects achieved through the physical encapsulation of rapamycin, the chemical conjugation of Ce nanoparticles and MEX in the CEX@Rapa hybrid systems. This unique combination harnesses the inflamed renal homing ability and immunoregulatory properties of MEX, the immunosuppressive effects of rapamycin, and the ROS scavenging capability of Ce nanoparticles. As a result, CEX@Rapa enhances immune modulation and alleviates renal complications in MRL/lpr mice. Collectively, our data demonstrate that intravenous administration of CEX@Rapa in the MRL/lpr mouse model significantly decreased both the occurrence and intensity of the disease, highlighting the probability of CEX@Rapa as a prophylactic therapy for LN.

## Conclusion

In conclusion, engineered MEXs offer a groundbreaking treatment strategy for LN. By developing a novel nanohybrid, CEX@Rapa, which integrates Ce nanoparticles with rapamycin-loaded MEXs, we have created a dual-function system capable of scavenging ROS while delivering targeted therapy. The exceptional characteristics of CEX@Rapa, including minimal toxicity, efficient drug loading, and remarkable cellular uptake, underscore its effectiveness as a therapeutic agent. The promising in vivo results demonstrate that CEX@Rapa preferentially accumulates in inflamed kidneys, where it exerts spatiotemporally coordinated actions: MEXs facilitate targeted delivery, elevated ROS triggers rapamycin release and activates Ce nanoparticles’ antioxidant function, and MEXs themselves contribute immunomodulatory and repair signals. This synergistic crosstalk among Ce nanoparticles (ROS scavenger), rapamycin (immunosuppressant), and MEXs (active regulator) enables potent modulation of the overactive immune response and facilitation of renal repair. This targeted delivery not only enhances therapeutic efficacy but also minimizes systemic side effects, a crucial consideration in treating chronic autoimmune conditions like LN. Given its synergistic antioxidant, anti-damage, and anti-inflammatory properties derived from Ce nanoparticles, MEX, and rapamycin, CEX@Rapa represents a significant advancement in the management of LN and other autoimmune diseases.

Despite these promising results, several aspects require further optimization to advance toward clinical translation. First, additional dose-response studies and dosing interval optimization are necessary to determine the optimal therapeutic window. Second, direct comparisons with standard-of-care therapies (e.g., mycophenolate mofetil and cyclophosphamide) will be essential to fully establish the relative efficacy and safety of CEX@Rapa. Third, large-scale manufacturing and reproducibility of engineered exosomes warrant further development to meet clinical-grade standards. Addressing these challenges will be critical to advancing the translation of engineered exosome-based therapies and achieving meaningful progress in the treatment of LN.

## Supplementary Information

Supplementary material 1

## Data Availability

No datasets were generated or analysed during the current study.

## References

[1] Hoi A, Igel T, Mok CC, Arnaud L. Systemic lupus erythematosus. Lancet. 2024;403(10441):2326–38. 38642569 10.1016/S0140-6736(24)00398-2

[2] De Vriese AS, Sethi S, Fervenza FC. Lupus nephritis: redefining the treatment goals. Kidney Int. 2025;107(2):198–211. 39521057 10.1016/j.kint.2024.10.018

[3] Tsokos GC. The immunology of systemic lupus erythematosus. Nat Immunol. 2024;25(8):1332–43. 39009839 10.1038/s41590-024-01898-7

[4] Taubmann J, Mueller F, Mutlu MY, Voelkl S, Aigner M, Bozec A, Mackensen A, Grieshaber-Bouyer R, Schett G. CD19 chimeric antigen receptor T cell treatment: unraveling the role of B cells in systemic lupus erythematosus. Arthritis Rheumatol. 2024;76(4):497–504. 38114423 10.1002/art.42784

[5] Krickau T, Naumann-Bartsch N, Aigner M, Kharboutli S, Kretschmann S, Spoerl S, et al. Cart-cell therapy rescues adolescent with rapidly progressive lupus nephritis from haemodialysis. Lancet. 2024;403(10437):1627–30. 38642568 10.1016/S0140-6736(24)00424-0

[6] Lai Z-W, Kelly R, Winans T, Marchena I, Shadakshari A, Yu J, Dawood M, Garcia R, Tily H, Francis L, Faraone SV, Phillips PE, Perl A. Sirolimus in patients with clinically active systemic lupus erythematosus resistant to, or intolerant of, conventional medications: a single-arm, open-label, phase 1/2 trial. Lancet. 2018;391(10126):1186–96. 29551338 10.1016/S0140-6736(18)30485-9 PMC5891154

[7] Huang NC, Winans T, Wyman B, Oaks Z, Faludi T, Choudhary G, et al. Rab4A-directed endosome traffic shapes pro-inflammatory mitochondrial metabolism in T cells via mitophagy, CD98 expression, and kynurenine-sensitive mTOR activation. Nat Commun. 2024. 10.1038/s41467-024-46441-2. 38519468 10.1038/s41467-024-46441-2 PMC10960037

[8] Mannick JB, Lamming DW. Targeting the biology of aging with mTOR inhibitors. Nat Aging. 2023;3(6):642–60. 37142830 10.1038/s43587-023-00416-y PMC10330278

[9] Schreiber KH, Apelo SIA, Yu D, Brinkman JA, Velarde MC, Syed FA, Liao C-Y, Baar EL, Carbaja KA, Sherman DS, Ortiz D, Brunauer R, Yang SE, Tzannis ST, Kennedyl BK, Lamming DW. A novel rapamycin analog is highly selective for mTORC1 in vivo. Nat Commun. 2019;10. 10.1038/s41467-019-11174-0 PMC6642166 31324799

[10] Burke JA, Zhang X, Bobbala S, Frey MA, Fuentes CB, Haddad HF, Allen SD, Richardson RAK, Ameer GA, Scott EA. Subcutaneous nanotherapy repurposes the immunosuppressive mechanism of Rapamycin to enhance allogeneic islet graft viability. Nat Nanotechnol. 2022;17(3):319. 35039683 10.1038/s41565-021-01048-2 PMC8934301

[11] Dilliard SA, Siegwart DJ. Passive, active and endogenous organ-targeted lipid and polymer nanoparticles for delivery of genetic drugs. Nat Rev Mater. 2023;8(4):282–300. 36691401 10.1038/s41578-022-00529-7 PMC9850348

[12] Song D, Zhao Y, Wang Z, Xu Q. Tuning lipid nanoparticles for RNA delivery to extrahepatic organs. Adv Mater. 2024;36:44. 10.1002/adma.202401445 PMC11530311 39233550

[13] Han J, Sheng T, Zhang Y, Cheng H, Gao J, Yu J, et al. Bioresponsive immunotherapeutic materials. Adv Mater. 2024;36:43. 10.1002/adma.202209778 36639983

[14] Han H, Li S, Xu M, Zhong Y, Fan W, Xu J, et al. Polymer- and lipid-based nanocarriers for ocular drug delivery: current status and future perspectives. Adv Drug Deliv Rev. 2023. 10.1016/j.addr.2023.114770. 36894134 10.1016/j.addr.2023.114770

[15] Li X, Wang S, Zhong J, Li T, Fan G, Zhou D, et al. Preparation and characterization of fine and stable short amylose nanocarriers for curcumin using a highly efficient and convenient method. Int J Biol Macromol. 2024. 10.1016/j.ijbiomac.2023.128738. 38092108 10.1016/j.ijbiomac.2023.128738

[16] Martinez JO, Molinaro R, Hartman KA, Boada C, Sukhovershin R, De Rosa E, Kirui D, Zhang S, Evangelopoulos M, Carter AM, Bibb JA, Cooke JP, Tasciotti E. Biomimetic nanoparticles with enhanced affinity towards activated endothelium as versatile tools for theranostic drug delivery. Theranostics. 2018;8(4):1131–45. 29464004 10.7150/thno.22078 PMC5817115

[17] Shang S, Li X, Wang H, Zhou Y, Pang K, Li P, et al. Targeted therapy of kidney disease with nanoparticle drug delivery materials. Bioact Mater. 2024;37:206–21. 38560369 10.1016/j.bioactmat.2024.03.014 PMC10979125

[18] Buzas EI. The roles of extracellular vesicles in the immune system. Nat Rev Immunol. 2023;23(4):236–50. 35927511 10.1038/s41577-022-00763-8 PMC9361922

[19] Fang Y, Ni J, Wang Y-S, Zhao Y, Jiang L-Q, Chen C, et al. Exosomes as biomarkers and therapeutic delivery for autoimmune diseases: opportunities and challenges. Autoimmun Rev. 2023. 10.1016/j.autrev.2022.103260. 36565798 10.1016/j.autrev.2022.103260

[20] Kalluri R, LeBleu VS. The biology, function, and biomedical applications of exosomes. Science. 2020;367:6478. 10.1126/science.aau6977 PMC7717626 32029601

[21] Deng D, Li X, Zhang J-J, Yin Y, Tian Y, Gan D, et al. Biotin-avidin system-based delivery enhances the therapeutic performance of MSC-derived exosomes. ACS Nano. 2023;17(9):8530–50. 37115712 10.1021/acsnano.3c00839

[22] Perets N, Betzer O, Shapira R, Brenstein S, Angel A, Sadan T, Ashery U, Popovtzer R, Offen D. Golden exosomes selectively target brain pathologies in neurodegenerative and neurodevelopmental disorders. Nano Lett. 2019;19(6):3422–31. 30761901 10.1021/acs.nanolett.8b04148

[23] Cao J-Y, Wang B, Tang T-T, Wen Y, Li Z-L, Feng S-T, Wu M, Liu D, Yin D, Ma K-L, Tang R-N, Wu Q-L, Lan H-Y, Lv L-L, Liu B-C. Exosomal miR-125b-5p deriving from mesenchymal stem cells promotes tubular repair by suppression of p53 in ischemic acute kidney injury. Theranostics. 2021;11(11):5248–66. 33859745 10.7150/thno.54550 PMC8039965

[24] Kumar MA, Baba SK, Sadida HQ, Marzooqi SA, Jerobin J, Altemani FH, et al. Extracellular vesicles as tools and targets in therapy for diseases. Signal Transduct Target Ther. 2024. 10.1038/s41392-024-01735-1. 38311623 10.1038/s41392-024-01735-1 PMC10838959

[25] Jiang H, Zhu X, Yu J, Wang W, Mao Y, Jiang L, et al. Biomimetic extracellular vesicles based on composite bioactive ions for the treatment of ischemic bone disease. ACS Nano. 2024. 10.1021/acsnano.4c13028. 39652362 10.1021/acsnano.4c13028

[26] Zhang M, Johnson-Stephenson TK, Wang W, Wang Y, Li J, Li L, et al. Mesenchymal stem cell-derived exosome-educated macrophages alleviate systemic lupus erythematosus by promoting efferocytosis and recruitment of IL-17^+^ regulatory T cell. Stem Cell Res Ther. 2022. 10.1186/s13287-022-03174-7. 36153633 10.1186/s13287-022-03174-7 PMC9509559

[27] Ma W, Che J, Chen W, Wang D, Zhang H, Zhao Y. Dexamethasone-integrated mesenchymal stem cells for systemic lupus erythematosus treatment via multiple immunomodulatory mechanisms. ACS Nano. 2024;18(20):13249–65. 38720584 10.1021/acsnano.4c02420

[28] Koo S, Sohn HS, Kim TH, Yang S, Jang SY, Ye S, Choi B, Kim SH, Park KS, Shin HM, Park OK, Kim C, Kang M, Soh M, Yoo J, Kim D, Lee N, Kim B-S, Jung Y, Hyeon T. Ceria-vesicle nanohybrid therapeutic for modulation of innate and adaptive immunity in a collagen-induced arthritis model. Nat Nanotechnol. 2023;18(12):1502–14. 37884660 10.1038/s41565-023-01523-y

[29] Kim YG, Lee Y, Lee N, Soh M, Kim D, Hyeon T. Ceria-based therapeutic antioxidants for biomedical applications. Adv Mater. 2024. 10.1002/adma.202210819. 36793245 10.1002/adma.202210819

[30] Han F, Tu Z, Zhu Z, Liu D, Meng Q, Yu Q, Wang Y, Chen J, Liu T, Han F, Li B. Targeting endogenous reactive oxygen species removal and regulating regenerative microenvironment at annulus fibrosus defects promote tissue repair. ACS Nano. 2023;17(8):7645–61. 37022700 10.1021/acsnano.3c00093

[31] Liu C, Gui L, Zheng J-J, Xu Y-Q, Song B, Yi L, et al. Intrinsic strain-mediated ultrathin ceria nanoantioxidant. J Am Chem Soc. 2023;145(34):19086–97. 37596995 10.1021/jacs.3c07048

[32] Kim J, Kim HY, Song SY, Go S-h, Sohn HS, Baik S, Soh M, Kim K, Kim D, Kim H-C, Lee N, Kim B-S, Hyeon T. Synergistic oxygen generation and reactive oxygen species scavenging by manganese Ferrite/Ceria Co-decorated nanoparticles for rheumatoid arthritis treatment. ACS Nano. 2019;13(3):3206–17. 30830763 10.1021/acsnano.8b08785

[33] Zhou F, Li M, Chen M, Chen M, Chen X, Luo Z, et al. Redox homeostasis strategy for inflammatory macrophage reprogramming in rheumatoid arthritis based on ceria oxide nanozyme-complexed biopolymeric micelles. ACS Nano. 2023;17(5):4358–72. 36847819 10.1021/acsnano.2c09127

[34] Huang Q, Yang Y, Zhao T, Chen Q, Liu M, Ji S, Zhu Y, Yang Y, Zhang J, Zhao H, Nan Y, Ai K. Passively-targeted mitochondrial tungsten-based nanodots for efficient acute kidney injury treatment. Bioact Mater. 2023;21:381–93. 36185743 10.1016/j.bioactmat.2022.08.022 PMC9483742

[35] Cordeiro RM. Reactive oxygen species at phospholipid bilayers: distribution, mobility and permeation. Biochim Biophys Acta. 2014;1838(1 Pt):438–44. 24095673 10.1016/j.bbamem.2013.09.016

[36] Liang R, Liu Y, Fu LM, Ai XC, Zhang JP, Skibsted LH. Antioxidants and physical integrity of lipid bilayers under oxidative stress. J Agric Food Chem. 2012;60(41):10331–6. 23016668 10.1021/jf3030979

[37] Zhu H, Huang D, Nie M, Zhao Y, Sun L. Dexamethasone loaded DNA scavenger nanogel for systemic lupus erythematosus treatment. Bioactive Mater. 2025;43:330–9. 10.1016/j.bioactmat.2024.08.030 PMC11923376 40115883

[38] Tseng W-C, Lee P-Y, Tsai M-T, Chang F-P, Chen N-J, Chien C-T, et al. Hypoxic mesenchymal stem cells ameliorate acute kidney ischemia-reperfusion injury via enhancing renal tubular autophagy. Stem Cell Res Ther. 2021. 10.1186/s13287-021-02374-x. 34183058 10.1186/s13287-021-02374-x PMC8240301

[39] Zhu H, Kong B, Nie M, Zhao C, Liu R, Xie Y, et al. ECM-inspired peptide dendrimer microgels with human MSCs encapsulation for systemic lupus erythematosus treatment. Nano Today. 2022. 10.1016/j.nantod.2022.101454.36570700

[40] Chen S, Saeed AFUH, Liu Q, Jiang Q, Xu H, Xiao GG, et al. Macrophages in immunoregulation and therapeutics. Signal Transduct Target Ther. 2023. 10.1038/s41392-023-01452-1. 37211559 10.1038/s41392-023-01452-1 PMC10200802

[41] Kopp JB, Anders H-J, Susztak K, Podesta MA, Remuzzi G, Hildebrandt F, et al. Podocytopathies. Nat Rev Dis Primers. 2020. 10.1038/s41572-020-0196-7. 32792490 10.1038/s41572-020-0196-7 PMC8162925

[42] Chiou TT-Y, Chau Y-Y, Chen J-B, Hsu H-H, Hung S-P, Lee W-C. Rapamycin attenuates PLA2R activation-mediated podocyte apoptosis via the PI3K/AKT/mTOR pathway. Biomed Pharmacother. 2021;144. 10.1016/j.biopha.2021.112349 34700229

[43] Zhou X-J, Klionsky DJ, Zhang H. Podocytes and autophagy: a potential therapeutic target in lupus nephritis. Autophagy. 2019;15(5):908–12. 30755075 10.1080/15548627.2019.1580512 PMC6526813

