# Supplementary material for: Engineered exosomes conferred with ROS-regulation and immuno-suppression for ameliorating lupus nephritis
Source: J Nanobiotechnology. 2025 Oct 10;23:639. doi: 10.1186/s12951-025-03731-1 (PMC12514823; doi:10.1186/s12951-025-03731-1)

**Supplementary**

**Engineered Exosomes Conferred with ROS-regulation and Immuno-suppression for Ameliorating Lupus Nephritis**

Jiang Tian^a^, Zexin Wang^b^, Zhicheng Tang^c^, Haofang Zhu^b,^*, and Lingyun Sun^a,c,^*

^a^ Department of Rheumatology and Immunology, Nanjing Drum Tower Hospital, Affiliated Hospital of Medical School, Nanjing University, Nanjing, China

^b^ Department of Rheumatology and Immunology, The First Affiliated Hospital of Anhui Medical University, Hefei, China

^c^ Department of Rheumatology and Immunology, The Second Affiliated Hospital of Anhui Medical University, Hefei, China

Email: [zhuhaofang@126.com](mailto:hfzhu@ahmu.edu.cn), [lingyunsun@nju.edu.cn](mailto:lingyunsun@nju.edu.cn)

**Methods**

**Materials:**

Cerium nitrate hexahydrate, citric acid and ammonia solution (28% in water) were kindly obtained from Aladdin Co., Ltd (Shanghai, China). 1,2-distearoyl-sn-glycero-3-phosphoethanolamine-*N*-[methoxy(polyethylene glycol)-2000] (DSPE-PEG), 1,2-distearoyl-*sn*-glycero-3-phosphoethanolamine-4-*N*-[maleimide(polyethylene glycol)-2000] (DSPE-PEG-MAL) and 1,2-distearoyl-*sn*-glycero-3-phosphoethanolamine-*N*-[Cyanine5(polyethylene glycol)-2000] (DSPE-PEG-Cy5) were purchased from Ponsure Biotechnology Co., Ltd (Shanghai, China). Tris-(2-carboxyethyl) phosphine (TCEP) hydrochloride was purchased from Invitrogen. Phosphate buffered solution (PBS, pH=7.4), Dulbecco’s modified eagle medium nutrient mixture F-12 (DMEM/F12) and Fetal bovine serum (FBS) were ordered from Gibco. Dulbecco’s modified eagle medium (DMEM) was commercially available from FuHeng Biology Co., Ltd (Shanghai, China). CCK-8 assay kit, Calcein AM, propidium iodide (PI), reactive oxygen species (ROS) assay kit and antifade mounting medium with DAPI were purchased from Beyotime Biotechnology Co., Ltd (Shanghai, China). Rapamycin (10 mM*1 mL in DMSO), puromycin aminonucleoside (PAN) and lipopolysaccharide (LPS) were obtained from MedChemExpress. Cy2-rapamycin and Cy5-rapamycin were custom ordered from Ruixi Biotechnology Co., Ltd (Xian China). Antibodies used for western blot, including CD81, TSG101, Alix, histone H10 and calnexin, were ordered from Boster Biological Technology Co., Ltd (Wuhan China). Antibodies used for flow cytometric analysis were FITC anti-CD86, APC anti-CD206, PE anti-F4/80, which obtained from Biolegend (Beijing China). Antibodies used for laser confocal microscopy analysis, including anti-CD68, anti-CD206, anti-iNOS, were obtained from Invitrogen. CellMask orange plasma membrane stain and alexa flour 568 goat anti-mouse IgG were also ordered from Invitrogen.

**1. Synthesis and functionalization of Ce NPs**

Cerium nitrate hexahydrate (0.25 mmol) and citric acid (0.5 mmol) were dissolved in 4 ml of deionized water. The mixture was rapidly introduced into 50 ml of a 0.4 M ammonia solution and stirred continuously for 24 hours at room temperature. The completion of the reaction was indicated by a color change from white to yellow. Subsequently, the solution underwent purification through dialysis for 24 hours using a 3000 MWCO dialysis bag, followed by centrifugation at 10,000g for 10 minutes. The resulting cerium nanoparticles (Ce NPs) were obtained through lyophilization.

For the purpose of water dispersion and maleimide functionalization, Ce NPs were encapsulated with phospholipid-polyethylene glycol (PEG) through the following procedure: 20 mg of DSPE-PEG2000 and 10 mg of DSPE-PEG2000-MAL were dissolved in 10 mL of chloroform, then mixed with 2 mL of chloroform-suspended Ce NPs at a concentration of 10 mg/mL. After 5-minute sonication, chloroform was completely removed via rotary evaporation followed by vacuum drying at 60 °C for 3 hours to ensure complete removal of the chloroform. Subsequently, the resultant sample was hydrated with 4 mL PBS under sonication to form a transparent colloidal suspension. The maleimide-functionalized cerium nanoparticles (Ce-Mal) were purified through centrifugation at 5,000g to remove excess PEG and filtered through a 0.22 µm membrane. Cy5-labelled Ce-Mal was prepared similarly by incorporating 1 mg of DSPE-PEG-Cy5 during the PEG encapsulation process.

**2. Extraction of exosomes from mesenchymal stromal cells**

Mesenchymal stromal cells-derived exosomes (MEXs) were extracted through ultracentrifugation methods as previously reported. Briefly, approximately 2.0×10^6^ mesenchymal stromal cells were seeded in a T75 culture flask. The cells were then cultured, and the supernatant was collected. Initially, debris were discarded by centrifugation at 300 g for 10 minutes, 2000 g for 10 minutes and 10,000 g for 30 minutes at 4 °C. Subsequently, the media were centrifuged at 100,000 g for 70 minutes using an XE-100 ultracentrifuge (Beckman). To further purify the sample, the MEXs pellet were re-suspended in 20mL of PBS and ultracentrifuged again at 100,000 g for 70 min minutes to remove the contaminating protein. Quantitative determination of proteins was carried out by a BCA assay kit (Beyotime). Exosomes markers, including CD81, TSG101, Alix, histone H10 and calnexin were analyzed by using the western blot method.

For fluorescence labeling, 100 µg of purified MSC-derived exosomes (1 mL) were incubated with 1 µL of 1 mM DiO (Vybrant™) dye in PBS at 37°C for 30 minutes. Following incubation, the mixture was ultracentrifuged at 120,000 × g for 70 minutes to remove excess unbound dye. The resulting pellet was washed and resuspended in PBS for subsequent use.

**3. Preparation and characterization of CEX@Rapa**

Rapamycin (Rapa) and MEXs were mixed in a 1:1 mass/mass ratio and sonicated using a XM-900T ultrasonic crusher. The settings used were 20% amplitude with six cycles of 30 seconds on and off, interspersed with a 2-minute cooling period between each cycle. To facilitate recovery of the MEXs membrane, the mixture was co-cultured at 37 °C for 4 hours. After washing with PBS through ultrafiltration (100 kDa MWCO) at 3000 rpm for 15 minutes, Rapa-loaded MEXs (MEX@Rapa) were obtained.

MEX@Rapa modified with Ce NPs (CEX@Rapa) was constructed through a chemical reaction between the MEX@Rapa as the core material and Ce NPs as the decorative elements. Maleimide-functionalized Ce NPs were conjugated to the surface of thiol-functionalized MEX@Rapa via the Michael addition reaction between thiol and maleimide groups. First, MEX@Rapa (100 μg/mL) suspended in 1 mL of PBS containing1 mM of TCEP (Invitrogen). The samples were incubated at 37 °C for 30 minutes. Following incubation, the cells were washed with PBS three times by ultrafiltration to obtain thiolated MEX-SH@Rapa. Subsequently, Ce-Mal diluted in PBS (200 μg /mL) was added to MEX-SH@Rapa, and the mixture was incubated for another 1 hour at 37 °C. During the incubation, the tubes were tapped every 5 minutes. Following the incubation, the sample were washed with PBS three times by ultrafiltration and the resulting CEX@Rapa were finally dispersed in PBS solution. Super-resolution microscopy data were obtained using a laser confocal microscopy (Leica, STELLARIS STED). For RT-qPCR quantification, total RNA of MEXs and CEX@Rapa was extracted using RNA-easy isolation reagent (Vazyme). The levels of mRNAs (including HMOX, CCL2, CCL5, COX-2, IL-6, IL-10, TGF-β and GAPDH) were determined by a QuantStudio 5 Real-time Instrument (Thermo Fisher Scientific).

The contents of Rapa loaded in CEX were quantified using a microplate reader (Spark, TECAN) at an absorption wavelength of 278 nm in PBS. The drug loading content (DLC) and drug loading efficient (DLE) were calculated using the following equation: DLC (%) = (weight of Rapa in CEX@Rapa/CEX@Rapa) × 100%; DLE (%) = (weight of Rapa in CEX@Rapa /weight of Rapa feeding) × 100%.

The zeta-potential, size distribution and morphology of these NPs were characterized and recorded through the Nanoparticle size and potential analyzer (NS-90Z Plus, OMEC) and Transmission electron microscope (HT7800, Htachi). The ultraviolet–visible absorption spectra were obtained using a using a microplate reader (Spark, TECAN).

**4. Western blotting**

Proteins of the MSCs and MEXs were extracted, and their concentrations were measured using a BCA Protein Assay Kit (Beyotime, Shanghai). The protein samples were lysed, then mixed with loading buffer and boiled at 95 °C for 10 minutes. The denaturated protein samples were loaded on 10% SDS-polyacrylamide gel. The electrophoresis was proceeded for 2 hours at 80 V, followed by the proteins transfer to a PVDF membrane at 220 mA for 80 minutes. The proteins on the membrane were blocked with 5% (w/v) skimmed milk for at least 1 hour with shake to prevent non-specific binding. Primary antibodies against CD81, TSG101, Alix, histone H10 and calnexin (all 1:1000) were applied overnight at 4 °C. The membranes were washed three times with TBST for 10 minutes, and incubated with the HRP-conjugated Goat Anti-Rabbit IgG second antibody (1:2000) for 1 h with shake. The results were performed using a ECL luminescent liquid (Servicebio) in a chemiluminescence imaging system (Tanon).

**5. SOD- and CAT-mimicking catalytic activity assay**

A Total Superoxide Dismutase Assay kit with WST-8 (Beyotime) was used to analyze the SOD-mimicking activity, following the instructions provided. Initially, 160 µl of WST/enzyme working solution was combined with 20 µl of Ce NPs, Ce-Mal, MEX, CEX, Rapa and CEX@Rapa in separate wells of a 96-well microplate. To start the SOD coupling reaction, 20 µl of reaction priming working solution was added. The mixture was incubated for 30 minutes at 37 °C, and then the absorbance at 450 nm was measured with a microplate reader (Spark, TECAN).

For the CAT-mimicking activity, the Catalase Assay Kit (Beyotime) was utilized. 40 µl of Ce NPs, Ce-Mal, MEX, CEX, Rapa and CEX@Rapa were mixed with 10 µl of H_2_O_2_ (1 mM). After reaction for 30 minutes at room temperature, 450 µl of stopping working solution was added to terminate the reaction by inverting the mixture. Subsequently, 200 µl of chromogen working solution was introduced, and the microplate was incubated for 15 minutes at room temperature in the dark. The absorbance at 520 nm was measured using a microplate reader (Spark, TECAN).

**6. In vitro Rapa release of CEX@Rapa**

To perform the in vitro Rapa release test, CEX@Rapa (equivalent to 100 µg Rapa) was suspended in 1 mL of PBS (pH 7.4) and placed in a dialysis bag (MWCO: 3000 Da). The bag was immersed in 20 mL of release medium (PBS, 1 mM H₂O₂, or 1 mM HOCl) and incubated at 37 °C under gentle agitation (70 rpm). At predetermined time intervals, 200 µL of the external release medium was withdrawn for analysis and replaced with an equal volume of fresh medium. The concentration of released rapamycin in the sampled medium was determined by measuring its UV absorbance at 278 nm using a microplate reader (Spark, TECAN), with a standard curve established for quantification.

**7. Cell cultures**

Raw 264.7 cells, purchased from American type culture collection (ATCC), were cultured in DMEM with 10% heat-inactivated FBS and 1% antibiotics at 37 °C in a humidified atmosphere with 5% CO_2_. Similarly, mouse podocyte clone-5 (MPC-5), also ordered from ATCC, were incubated in DMEM/F12 with 10% heat-inactivated FBS and 1% antibiotics under the same conditions Both two cell lines have been authenticated and reliably used in multiple labs without any issues.

**8. Cellular uptake study**

The RAW264.7 cells were seeded onto 6-well plates at a density of 2🞨10^5^ cells per well, and cultured for 24 h in the absence or presence of LPS (10 μg/mL). Subsequently, the cells were treated with Cy2-labelled Rapa (5 ug/ml) and cy2-labelled CEX@Rapa (21 µg/mL, Rapa-equivalent) for 2 hours, followed by washing three times with PBS (pH 7.4). The cells were stained with DAPI for 10 min, and internalization was observed using a laser confocal microscopy (Leica, STELLARIS STED). To quantify the internalization, the same seeding and treatment procedures were repeated. After triple washing with PBS (pH 7.4), the cells were collected and the fluorescence intensity was assessed using a DxPAthenaM flow cytometry (Cytek). Similarly, MPC-5 cells were seeded onto 6-well plates and cultured for 24 h either with or without PAN (15 μg/mL). Then, the cells were treated with Cy5-labelled Rapa and CEX@Rapa for 2 h, followed by washing three times with PBS (pH 7.4). The cells were fixed with 4% paraformaldehyde for 20 minutes. After washing three time by PBS, the cells were stained overnight with Actin Green 488 (Invitrogen) at 4 ℃. Following this, the cells were stained with DAPI for 10 minutes and examined using a laser confocal microscopy (Leica, STELLARIS STED). The fluorescence intensity was also quantified by a DxPAthenaM flow cytometry (Cytek).

**9. ROS-scavenging effect of CEX@Rapa on macrophages**

RAW 264.7 cells were seeded in 6-well plates and treated with different formulations for 2 hours at their respective effective concentrations: 5 µg/mL for free Rapa, 21 µg/mL for MEX, CEX, and CEX@Rapa, and 8 µg/mL for Ce NPs (a carrier-equivalent concentration that delivers an equivalent dose of 5 µg/mL Rapa in the CEX@Rapa group). Following by washing the cells three times with PBS, the cells were treated with 0.1 mM H_2_O_2_ (Sigma Aldrich) for 45 minutes to induce temporary reactive oxygen species (ROS) overproduction. After three additional PBS washes, the cells were incubated with 10 µM DCFH-DA (Beyotime) fluorescent dye for 30 minutes while cells were kept in the dark. Cellular ROS levels were observed under a laser confocal microscopy (Leica, STELLARIS STED) and quantified by ImageJ software.

The percentage of ROS-positive cells was calculated by dividing the number of DCF-fluorescent cells (N_positive) by the total cells counted in bright-field images (N_total), averaged across ≥5 independent images per group. ROS Positive Cells (%) = (N_positive / N_total) × 100%

**10. Intracellular ROS detection of MPC-5 cells**

The effects of intracellular ROS level were assessed via a DCFH-DA probe. After seeding MPC-5 cells for 24 h, the cells were treated by PAN with 15 µg/mL for 24 hours. Subsequently, the cells were exposed to different formulations for 4 hours at their respective effective concentrations: 5 µg/mL for free Rapa, 21 µg/mL for MEX, CEX, and CEX@Rapa, and 8 µg/mL for Ce NPs and Ce-Mal. Following incubation with the cells with 10 μM DCFH-DA, the fluorescent signals of the cells were acquired using a DxPAthenaM flow cytometry (Cytek).

**11. Polarization of M1-to-M2 macrophages**

The macrophages were seeded onto 6-well plates and treated with LPS (10 µg/mL) for 24 hours, followed by exposure to various formulations for an additional 48 hours at their respective effective concentrations: 5 µg/mL for free Rapa, 21 µg/mL for MEX, CEX, and CEX@Rapa, and 8 µg/mL for Ce NPs and Ce-Mal. After washing three times with PBS, the cells were fixed with 4% paraformaldehyde for 20 minutes. the cells were then permeabilized with 0.5% Triton X-100 (Biosharp) for 10 minutes and blocked with 5% FBS for 15 minutes. Subsequently, the cells were stained overnight with primary antibody (iNOS, CD206, CD68) at 4 ℃. Afterward, the cells were incubated with a secondary antibody for 1 hours at room temperature and were stained with DAPI for 10 minutes. Between each step, the cells underwent three washes with PBS. Finally, the polarization of M1-to-M2 macrophages was observed using a laser confocal microscopy (Leica, STELLARIS STED) and the quantitative resulting were analyzed by DxPAthenaM flow cytometry (Cytek).

**12. Repairing of the damaging MPC-5 cells**

MPC-5 cells were seeded onto 6-well plates and cultured for 24 h in the absence or presence of PAN (15 μg/mL). Subsequently, the cells were exposed to different formulations for 24 or 48 hours at their respective effective concentrations: 5 µg/mL for free Rapa, 21 µg/mL for MEX, CEX, and CEX@Rapa, and 8 µg/mL for Ce NPs and Ce-Mal. To analyze the recovery of renal cells, the cytoskeleton was stained with the microfilament specific dye Actin Green 488. The proliferation of the injured renal cells was evaluated using a CCK-8 assay kit (Beyotime).

**13. In vivo evaluation of SLE treatment**

A mouse model of systemic lupus erythematosus (SLE)-like disease (MRL/lpr mice, male, specific pathogen-free (SPF), 10-12 weeks old, sourced from SiPeiFu, Beijing, China) was established to evaluate the in vivo therapeutic effects of NPs, given that MRL/lpr mice would spontaneously develop SLE over several months. All in vivo experiments adhered to the Ethics Committee of Drum Tower Hospital, with approval from its Laboratory Animal Welfare Ethics Committee of Drum Tower Hospital (Approval No. 2021AE01008). Following the successful establishment of the SLE model, treatments including 200 μL of 0.9% NaCl, Ce NPs, MEX, CEX, Rapa, and CEX@Rapa were administered via tail vein injection to the MRL/lpr mice once weekly for four weeks (weeks 15 to 18), within thirty MRL/lpr mice were evenly divided into five groups. Body weights were recorded at each treatment session. Mesangial cell density was quantified by ImageJ software in random areas of glomeruli. Quantitative measurements of fluorescence intensity in the glomeruli were also performed using ImageJ, which involved calculating the areas stained red (IgG) and green (C3). Creatinine levels in mice serum were analyzed using a creatinine assay kit (Jiancheng), and blood urea nitrogen (BUN) levels were tested by a BUN assay kit (Jiancheng Bioengineering). Anti-dsDNA Abs, TNF-α and IL-6 levels in mice serum were detected by ELISA kit (Multi sciences).

**14. Biodistribution of materials**

MRL/lpr mice were intravenously injected with Cy5 labeled Rapa and DiD labeled CEX@Rapa to evaluate biodistribution. Briefly, 100 µg of CEX@Rapa (1 mL) was incubated with 1 µL of 1 mM DiD (Vybrant™) dye in PBS at 37℃ for 30 minutes. Excess dye was removed by ultracentrifugation at 120,000 × g for 70 minutes. The biodistribution of the NPs was then monitored by near-infrared fluorescence (NIRF) imaging using an AniView 100 in vivo imaging scanner (Biolight Biotechnology) at different time points over 24 hours. At 2-, 4-, and 24-hours, one mouse from each group was sacrificed for dissection. The lymph nodes, heart, lung, liver, kidney, and spleen were taken out for ex vivo NIRF imaging and their mean NIRF intensity was calculated by AniView software. Additionally, fresh lymph nodes, heart, lung, liver, kidney, and spleen were frozen and sectioned using an HM525 NX automatic cryostat (Epredia). The sections were stained with DAPI for 10 minutes and observed using a laser confocal microscopy (Leica, STELLARIS STED).

**15.** **Safety evaluation**

For organ toxicity assessments, twenty-five healthy mice were randomly assigned to five groups: 0.9% NaCl, Ce NPs, MEX, CEX, Rapa, CEX@Rapa, with all doses were 1 mg/kg. The 0.9% NaCl solution or the respective drug was injected via the tail vein of the mice once a week for a total of four injections. In the fourth week, the animals were sacrificed and samples were collected, including serum and major organs. The serum was used to analyze liver and kidney function. The tissues were cleaned, fixed, sliced, and subjected to hematoxylin-eosin staining, after which the histological changes were visualized and captured using a KF-PRO-020 Digital Pathology Slide Scanner (KFBIO).


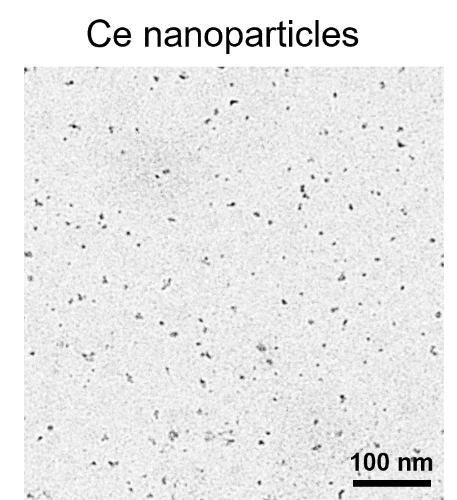


**Figure S1.** TEM image of Ce nanoparticles.


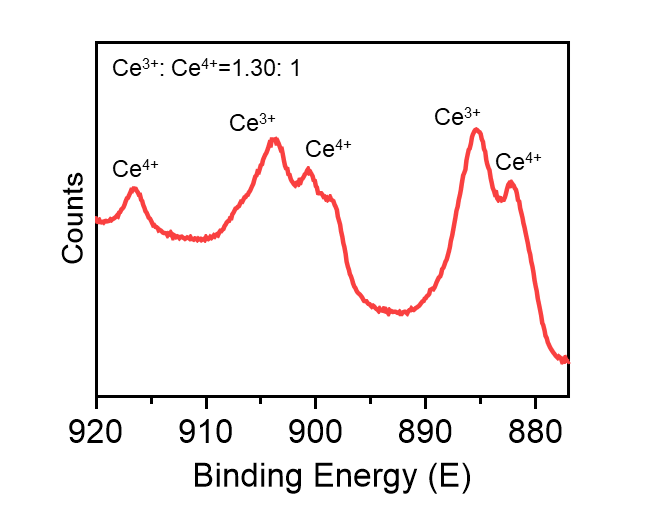


**Figure S2.** The Ce³⁺/Ce⁴⁺ ratio using X-ray photoelectron spectroscopy.

**
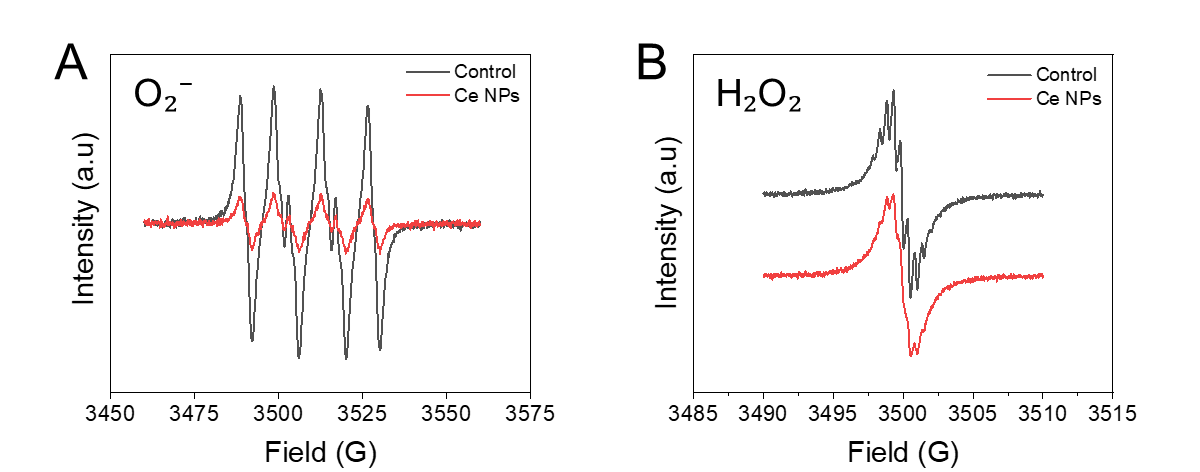
**

**Figure S3**. O₂⁻ and H₂O₂ scavenging performance using EPR spectroscopy.


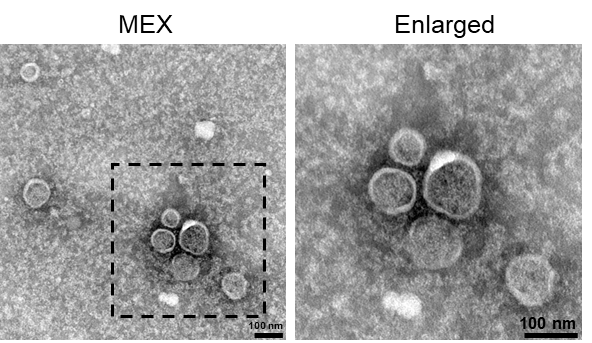


**Figure S4.** TEM image of MEX.

**
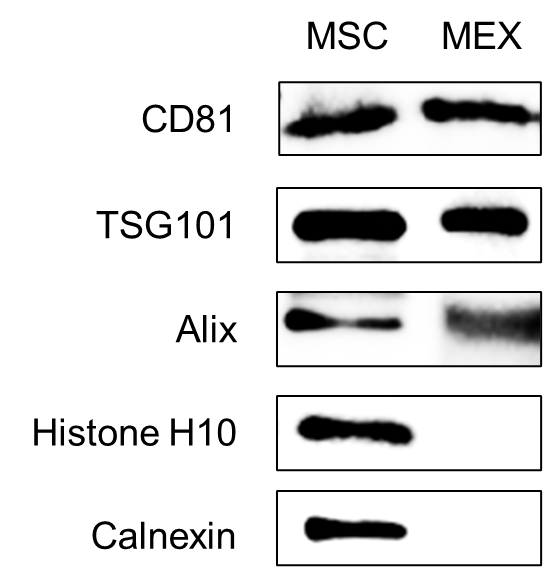
**

**Figure S5.** Western blot analysis evaluating protein profiles of both MSCs and MEX.

**
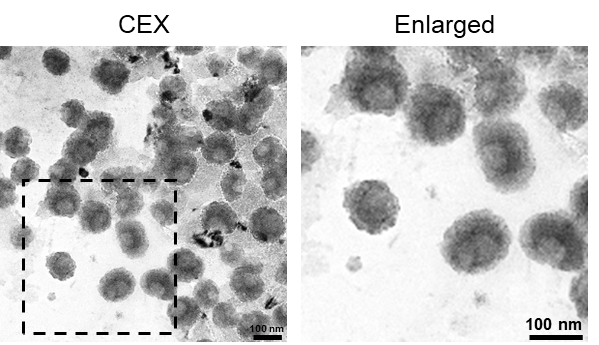
**

**Figure S6.** TEM image of CEX.

**
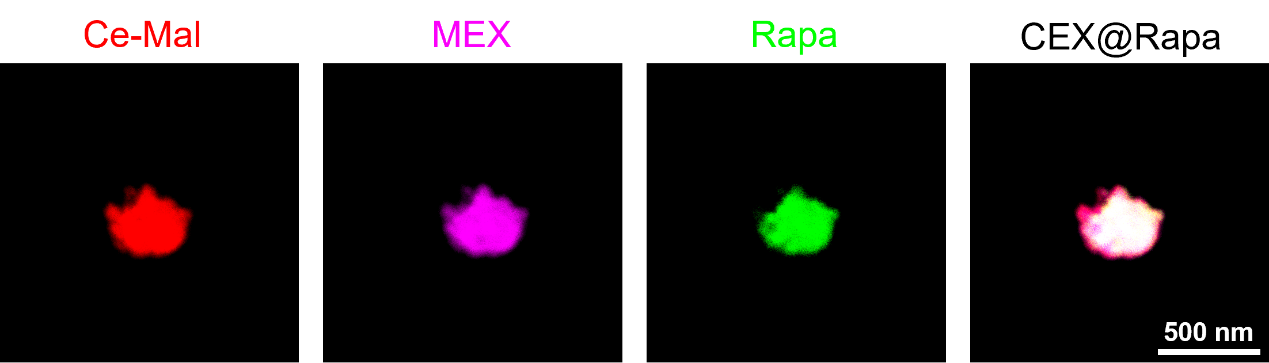
**

**Figure S7.** The co-localization of Cy5-labeled Ce-Mal (red), DiO-labeled MEX (violet), and Cy2-labeled rapamycin (green) shown in super-resolution microscopy images.


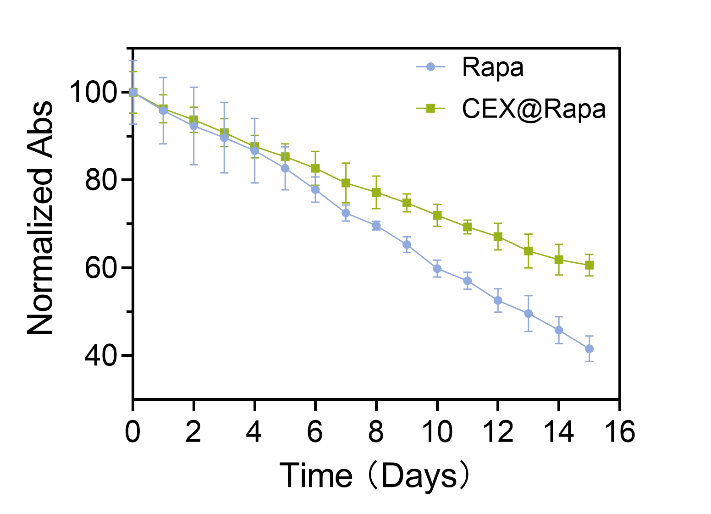


**Figure S8.** UV spectra showing the stability of free rapamycin and rapamycin loaded in the CEX@Rapa nanohybrids.

**
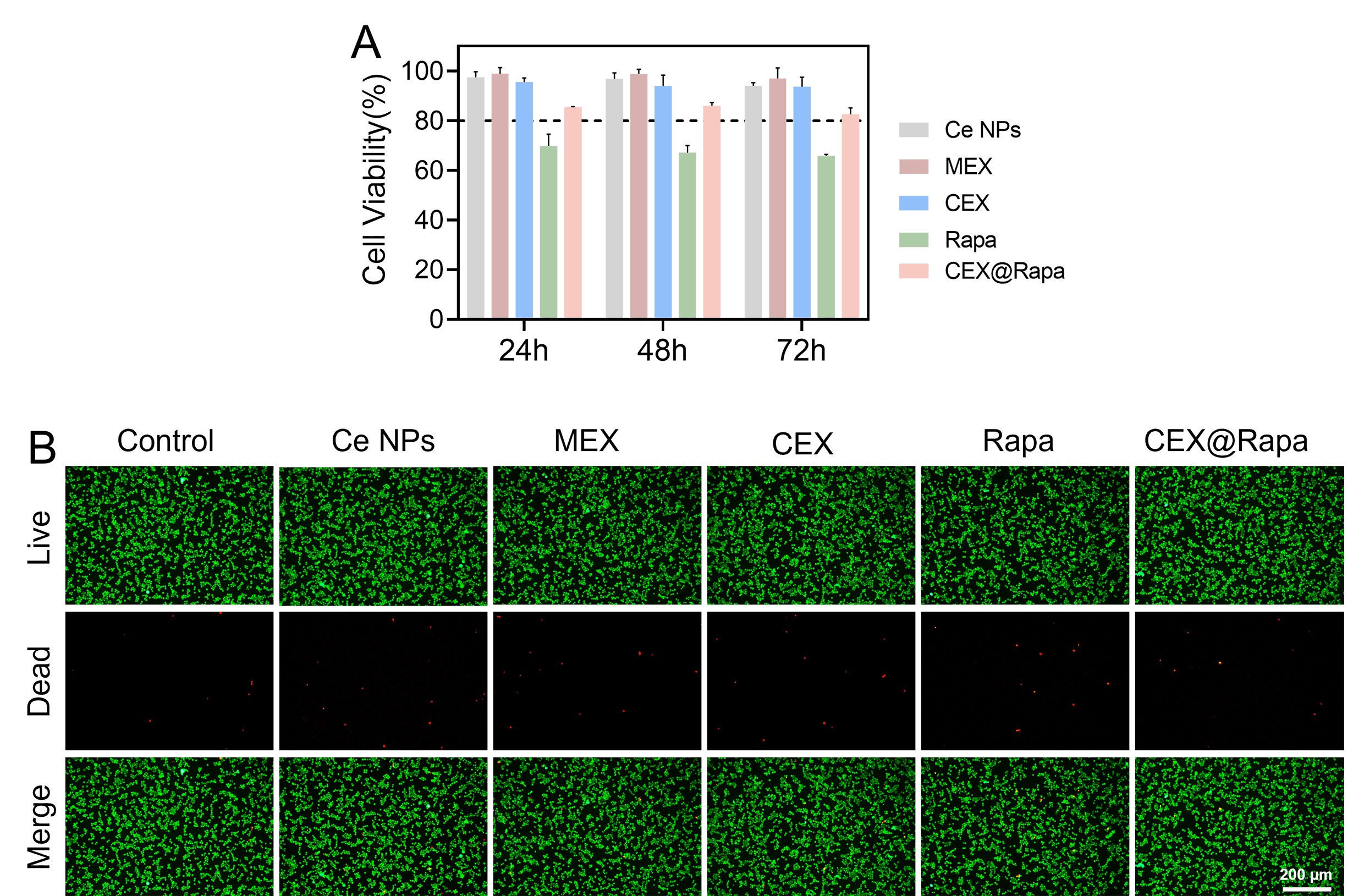
**

**Figure S9**. The biocompatibility of Ce nanoparticles, MEX, CEX, rapamycin, and CEX@Rapa evaluated by (A) CCK-8 assay and (B) LIVE/DEAD staining on RAW 264.7 cells.


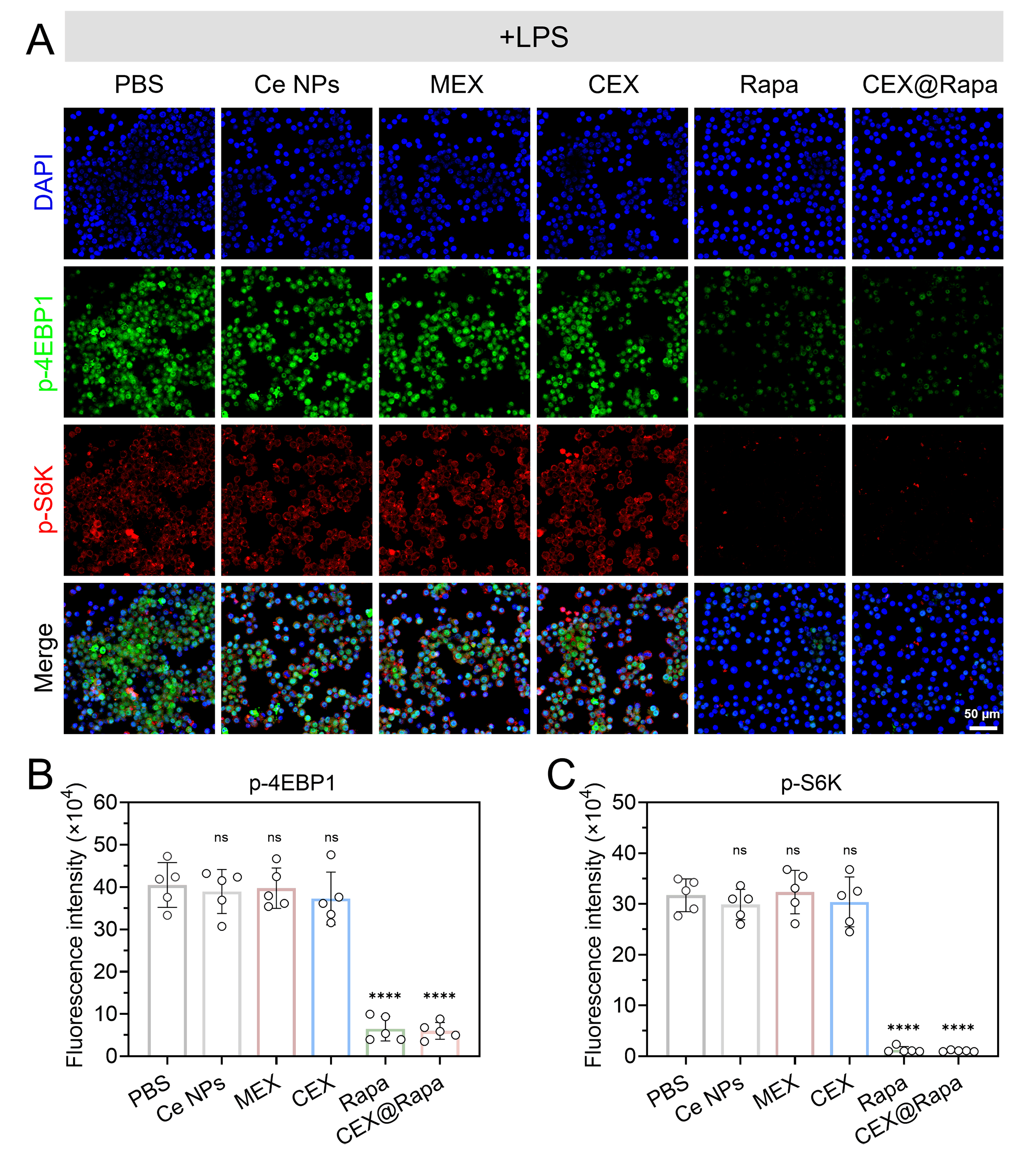


**Figure S10**. Immunofluorescence analysis of mTOR inhibition. (A) Representative immunofluorescence images of macrophages stained for p-4EBP1 (green), p-S6K (red). Nuclei (DAPI, blue). (B, C) Quantitative analysis of fluorescence intensity of p-S6K and p-4EBP1. Data are presented as mean ± standard deviation (n = 5), with statistical significance evaluated relative to the PBS group using one-way ANOVA and Tukey’s post hoc test. ****P < 0.0001. ns, not significant.


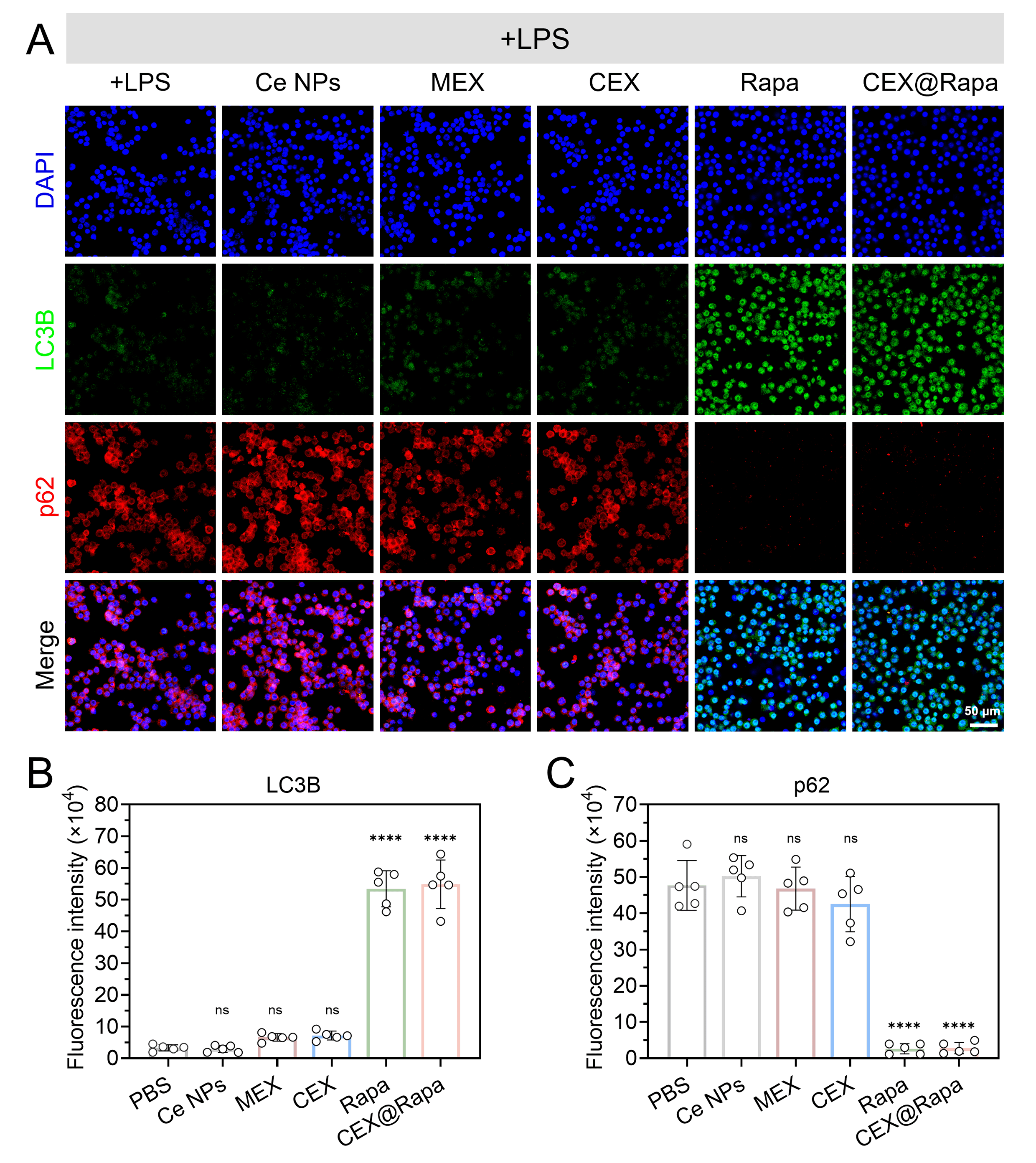


**Figure S11**. Immunofluorescence analysis of autophagy flux. (A) Representative immunofluorescence images of macrophages stained for LC3B (green), p62 (red). Nuclei (DAPI, blue). (B, C) Quantitative analysis of fluorescence intensity of LC3B and p62. Data are presented as mean ± standard deviation (n = 5), with statistical significance evaluated relative to the PBS group using one-way ANOVA and Tukey’s post hoc test. ****P < 0.0001. ns, not significant.


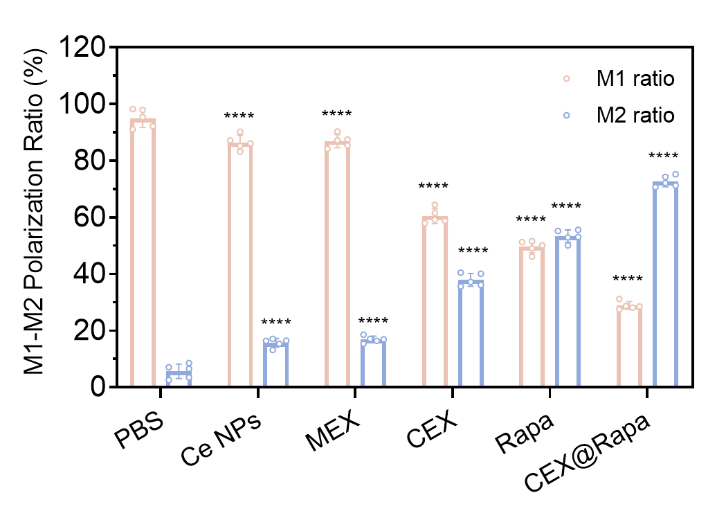


**Figure S12**. M1-M2 polarization ratio after treatment. Data are presented as mean ± standard deviation (n = 5), with statistical significance evaluated relative to the Control group using one-way ANOVA and Tukey’s post hoc test. ****P < 0.0001.


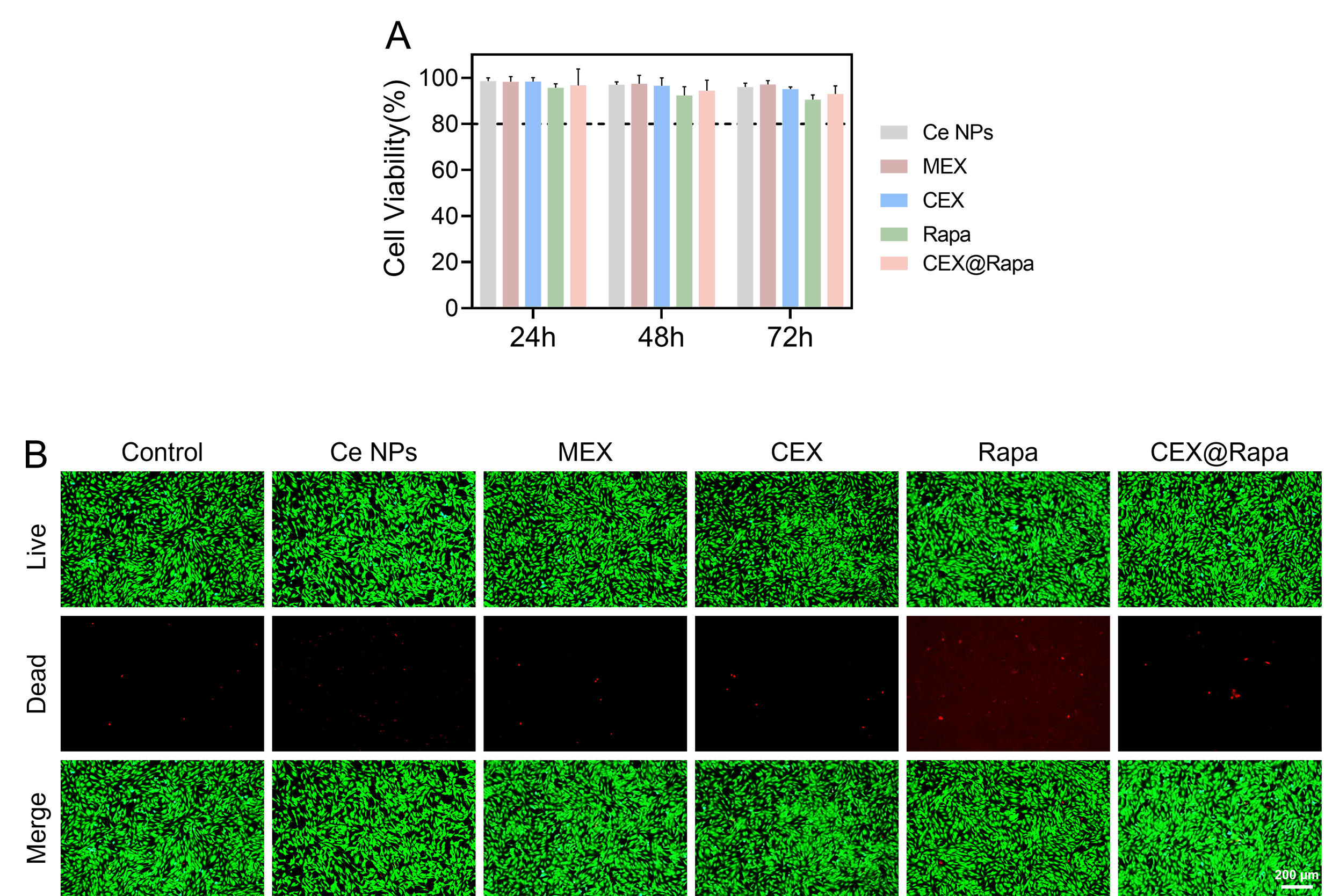


**Figure S13**. The biocompatibility of Ce nanoparticles, MEX, CEX, rapamycin, and CEX@Rapa evaluated by (A) CCK-8 assay and (B) LIVE/DEAD staining on MPC-5 cells.


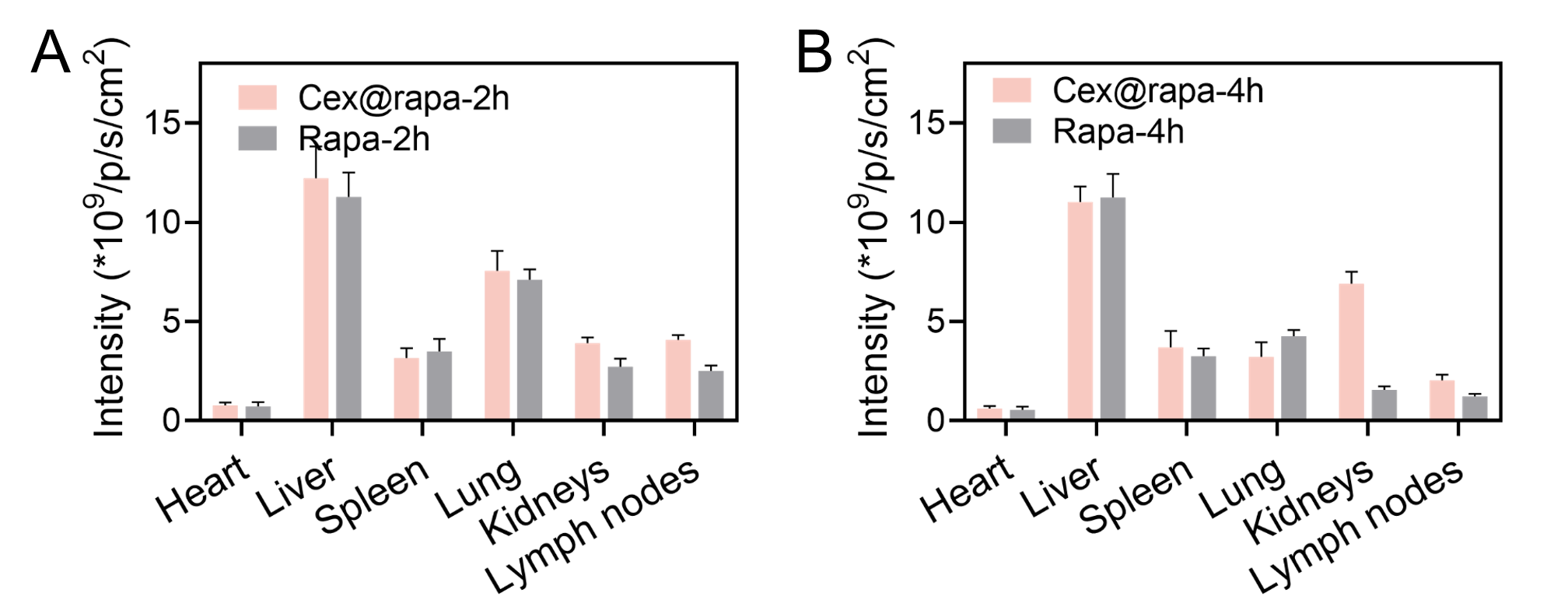


**Figure S14**. Ex vivo distribution study analyzed average NIRF intensity on MRL/lpr mice, with measurements taken 2 and 4 hours post-intravenous injection.


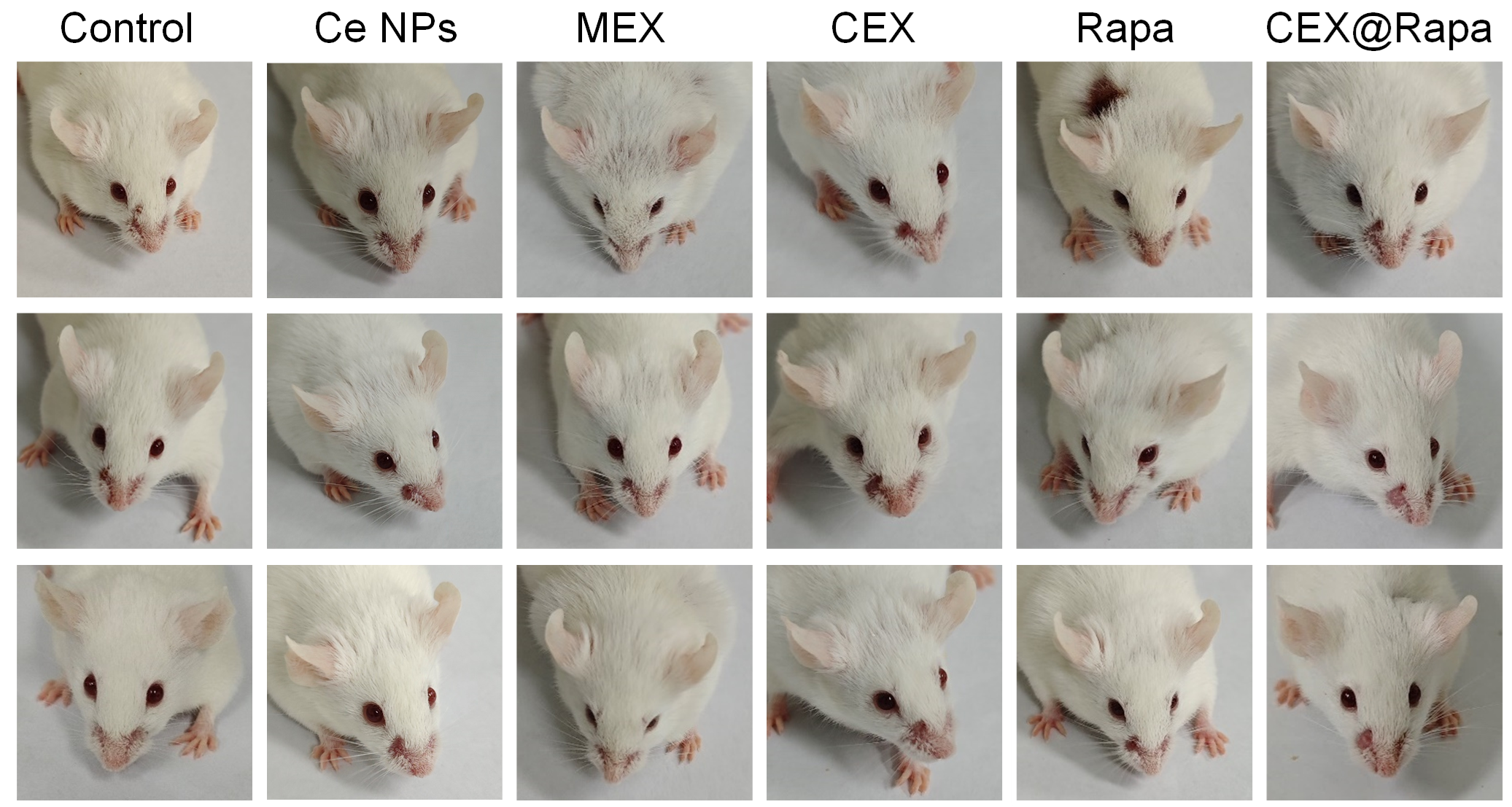


**Figure S15**. Facial lesions on the faces of mice at week 15 in PBS, Ce nanoparticles, MEX, CEX, rapamycin, and CEX@Rapa groups.


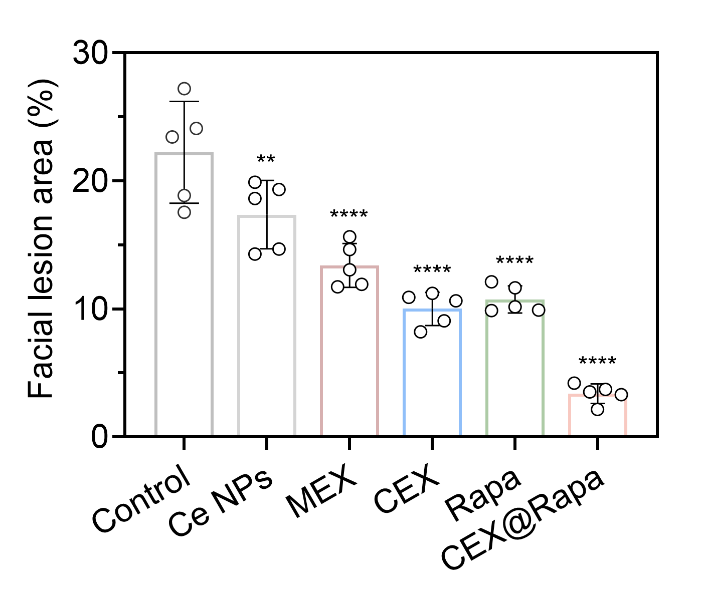


**Figure S16**. Quantitative analysis of mice facial lesions severity at week 20. Data are presented as mean ± standard deviation (n = 5), with statistical significance evaluated relative to the Control group using one-way ANOVA and Tukey’s post hoc test. **0.001 < P < 0.01, ****P < 0.0001. ns, not significant.


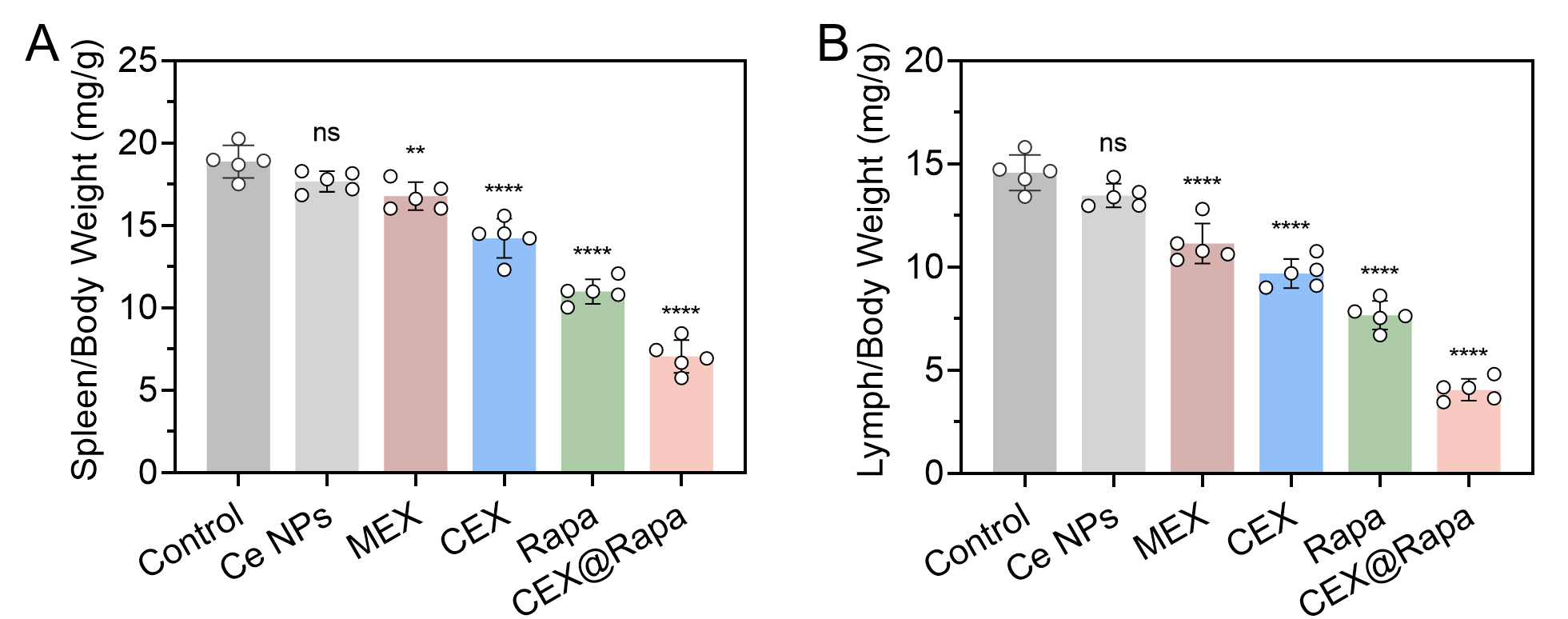


**Figure S17**. Comparative spleen and lymph nodes weight across different treatment groups. Data are presented as mean ± standard deviation (n = 5), with statistical significance evaluated relative to the Control group using one-way ANOVA and Tukey’s post hoc test. ****P < 0.0001. ns, not significant.

**Figure S18**. Body weight of MRL/lpr mice during treatment


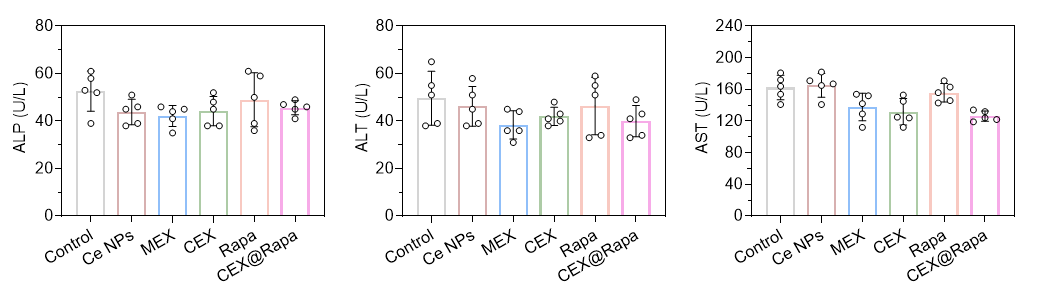


**Figure S19.** Safety assessment of liver function (ALP, ALT, and AST).


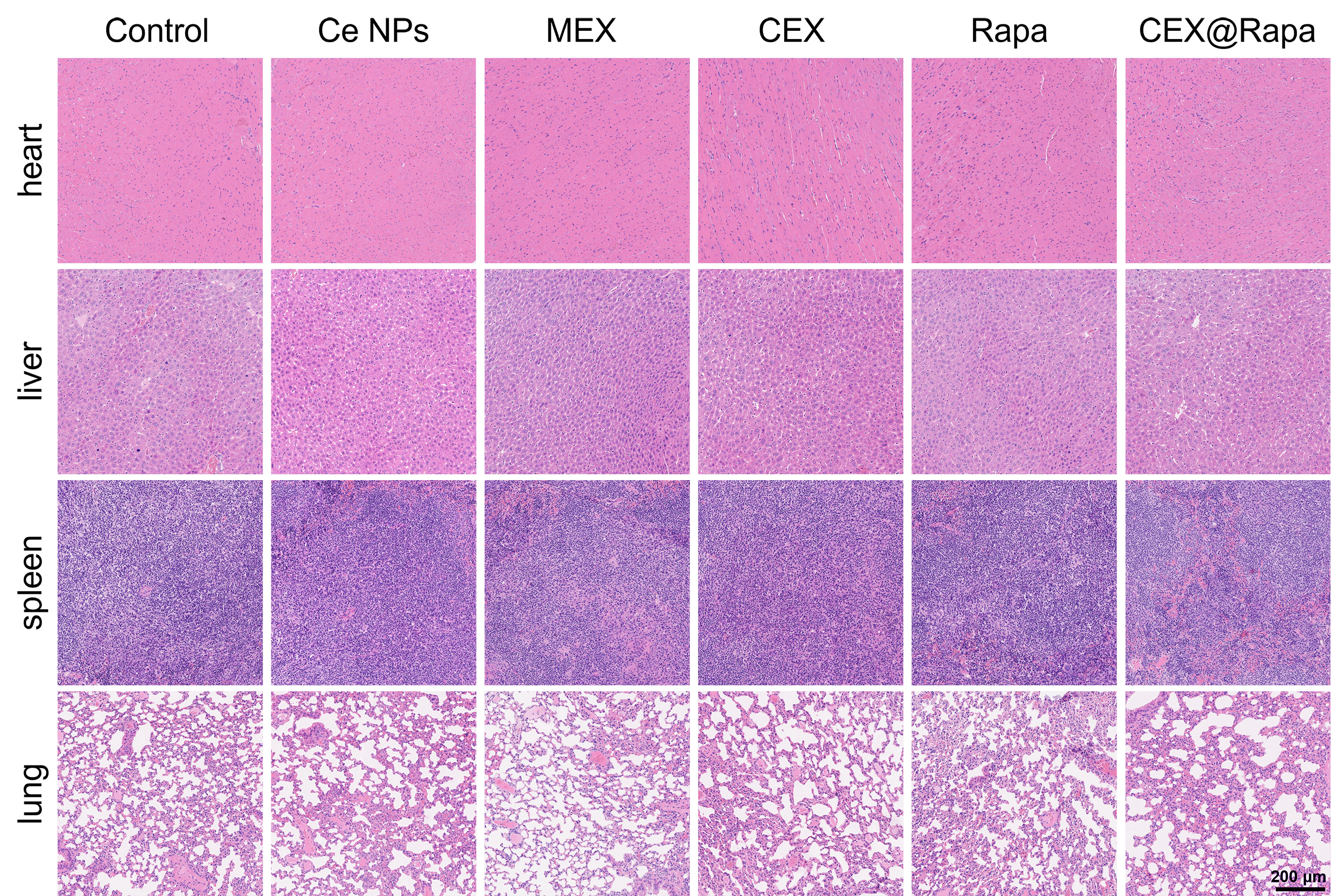


**Figure S20.** H&E dyeing of major organs from MRL/lpr mice in the PBS, Ce nanoparticles, MEX, CEX, rapamycin, and CEX@Rapa groups.

**Table S1. Quality control metrics of exosomes**

(Data represent mean from three independent batches prepared by ultracentrifugation)


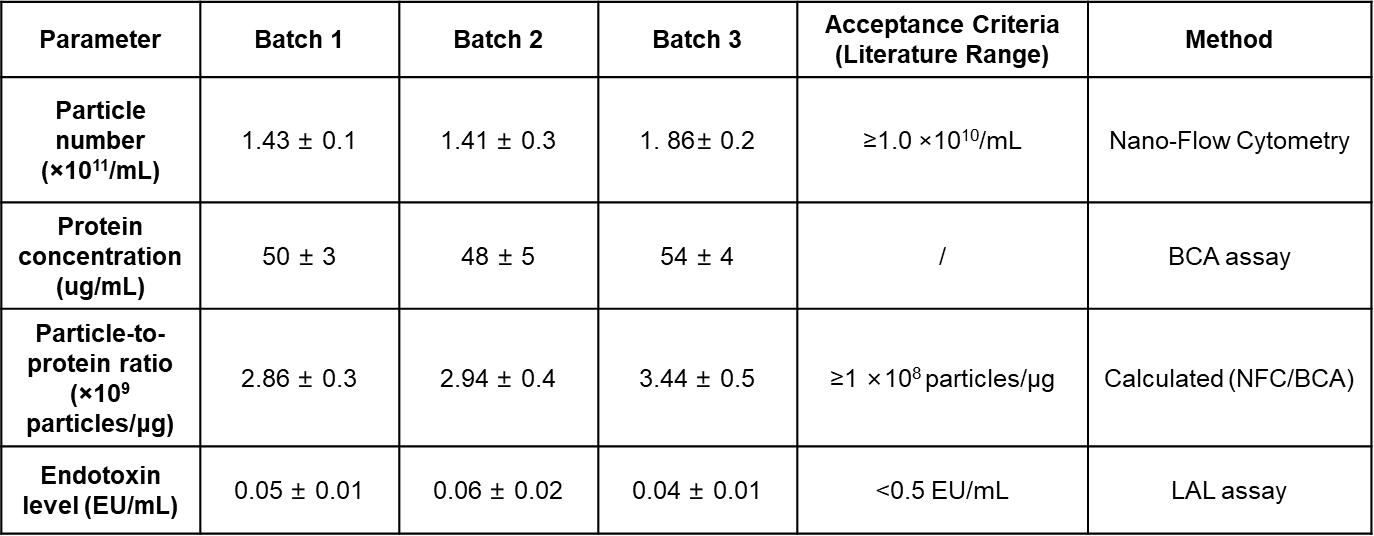

Supplement: Supplementary file 1 — Supplementary material 1 [file 12951_2025_3731_MOESM1_ESM.docx]
